# Doubly Stereogenic Sandwich Frameworks: Diastereomeric Metallobiscorroles

**DOI:** 10.1021/acs.inorgchem.5c00598

**Published:** 2025-05-05

**Authors:** Kristian Torstensen, Florian Sixt, Abraham B. Alemayehu, Nicholas S. Settineri, Abhik Ghosh

**Affiliations:** a Department of Chemistry, University of Tromsø, Tromsø N-9037, Norway; b Advanced Light Source, Lawrence Berkeley National Laboratory, Berkeley, California 94720-8229, United States

## Abstract

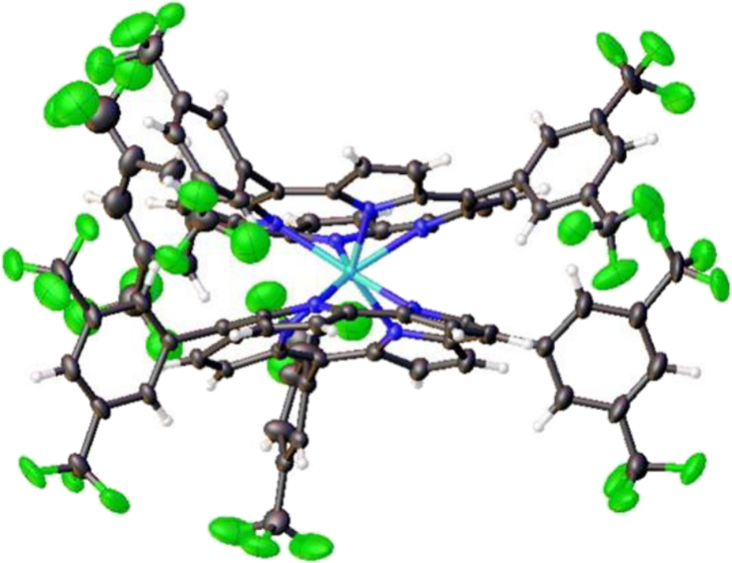

A number of Group 6 metallobiscorrole sandwich compounds with square-antiprismatic coordination were separated into diastereomers by means of careful preparative thin-layer chromatography. The diastereomers differ with respect to the relative orientation of the corrole macrocycles, which are rotated approximately ± 45° or ± 135° relative to each other. The most clear-cut results were obtained for two tungsten corroles, W[TBCF_3_PC]_2_ {TBCF_3_PC = *meso*-tris[3,5-bis(trifluoromethyl)phenyl]corrolato} and W[TDOMePC]_2_ [TDOMePC = *meso*-tris(3,5-dimethoxyphenyl)corrolato], for which single-crystal X-ray structures were obtained for the 135° diastereomer; the existence of the 45° diastereomer was inferred by elimination and with support from DFT calculations. For Mo[TBCF_3_PC]_2_ and W[TBCF_3_PC]_2_, both diastereomers were also fully characterized spectroscopically and their ^1^H NMR spectra were essentially fully assigned. The fact that each diastereomer is chiral and exists as two enantiomers (which was previously demonstrated for the 135° form of a tungsten biscorrole) establishes the doubly stereogenic nature of the metallobiscorrole framework – to our knowledge, the first such demonstration for a sandwich compound.

## Abstract 2

A number of Group 6 metallobiscorrole sandwich compounds with square-antiprismatic coordination were separated into diastereomers by means of careful preparative thin-layer chromatography.

## Introduction^1^

Sandwich compounds involving porphyrin, phthalocyanine and related ligands have been known for decades.^2^ Classic neutral complexes typically involve a +IV central metal ion such as Zr(IV) and Hf(IV)^3,4^ and even early actinides such as Th(IV) and U(IV).^5^ A wide range of lanthanide-porphyrinoid sandwich compounds are also known. In general, the metal ion exhibits square-antiprismatic coordination, which translates to an idealized *D*_4d_ symmetry for homoleptic complexes. Homoleptic corrole sandwich compounds are of much more recent provenance (the coordination chemistry of corroles has been has been the subject of several recent reviews^6−10^) and the best-characterized examples involve Mo(VI) and W(VI) as central ions.^11,12^ Rhenium biscorrole sandwich compounds have also been reported, albeit with only EXAFS and other spectroscopic evidence.^13^ Because of the lower symmetry of the corrole macrocycle, metallobiscorroles with square-antiprismatic coordination exhibit at best *C*_2_ symmetry; these species, accordingly, are chiral. Indeed, tungsten biscorroles have been resolved into enantiomers via HPLC on a chiral stationary phase and the enantiomers have been characterized via electronic circular dichroism spectroscopy.^14,15^ A key conclusion from this exercise was that the square-antiprism framework is configurationally stable, with the corroles essentially locked in place and unable to rotate relative to each other. DFT calculations also provided ample support to such a conclusion.^11^

**Scheme 1:**
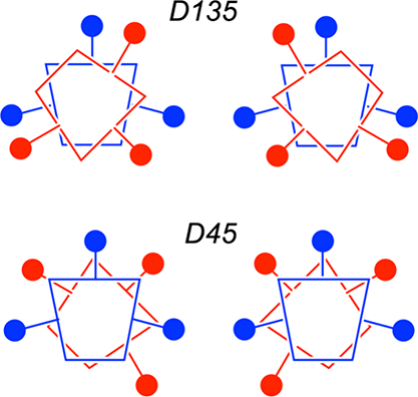
Metallobiscorroles as Doubly Stereogenic Frameworks

As it happens, there is a further twist to the stereochemistry of metallobiscorroles. The framework may be described as *doubly stereogenic*, leading to both diastereomerism and enantiomerism and a total of four stereoisomers.^16^ Square-antiprismatic coordination may leave the two corroles rotated either approximately ± 45° or ± 135° relative to each other, leading to two diastereomers, which we will denote hereafter as D45 and D135. In addition, each diastereomer is chiral and thus must exist as a pair of enantiomers (Scheme 1). To date, all structurally characterized metallobiscorroles, however, have been found to exhibit the D135 stereochemistry.^11−13^ DFT calculations on peripherally unsubstituted metallobiscorroles, however, suggest that both diastereomers are comparably stable in a thermodynamic sense.^11^ The question as to whether the D45 diastereomer may be synthetically accessible has remained an open one, until now.

Although high-quality X-ray structures have been obtained for several metallobiscorroles,^11−13^ their ^1^H NMR spectra have remained utterly indecipherable, with extensive signal overlap in the aromatic region. The present study, as it happens, began with the humble goal of identifying corrole ligands that would lead to less cluttered ^1^H NMR spectra. Working with *meso*-tris(pentafluorophenyl)corrole (H_2_[TFPPC]),^17,18^*meso*-tris(3,5-dimethoxyphenyl)corrole (H_2_[TDOMePC]),^19^ and *meso*-tris[3,5-bis(trifluoromethyl)phenyl]corrole (H_2_[TBCF_3_PC]),^18^ we uncovered not only several cases of well-resolved NMR spectra, but also, to our surprise, persuasive evidence of the presence of metallobiscorrole diastereomers.

## Results and Discussion

**Scheme 2:**
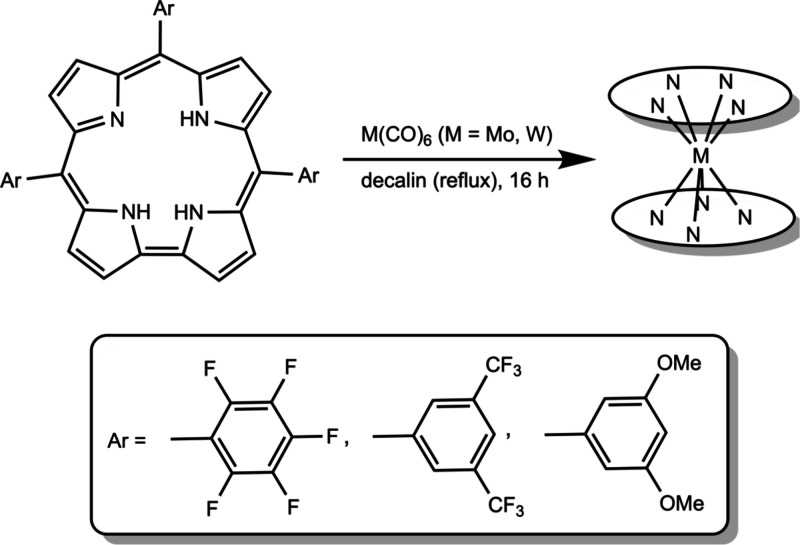
Optimized Synthetic Route to Substituted Metallobiscorroles

Our attempts to synthesize metallobiscorroles based on the TPFPC, TDOMePC and TBCF_3_PC ligands began with a number of setbacks. The literature procedure,^11^ involving the interaction of the free-base corroles with Group 6 hexacarbonyls (M = Mo, W) and K_2_CO_3_ in refluxing decalin yielded minute quantities of the desired sandwich compounds. Switching K_2_CO_3_ to 2,6-lutidine or DBU proved entirely ineffectual as did longer reaction times. (The nitrogen bases did effect corrole deprotonation, as judged by changes to the optical spectra, but failed to yield the desired products. Thus, K_2_CO_3_ appears to serve not merely as a base, but rather as a critical reactant, in an as-yet-undefined manner.) Adding multiple batches of M(CO)_6_, however, resulted in greatly improved yields, generally on the order of 10%, but >40% in the case of W[TBCF_3_PC]_2_ (Scheme 2). Attempts to synthesize biscorroles with 2,6-disubstituted *meso*-aryl groups, on the other hand, led to yields of only about 1%, underscoring the sensitivity of the reaction to steric effects.

**Figure 1:**
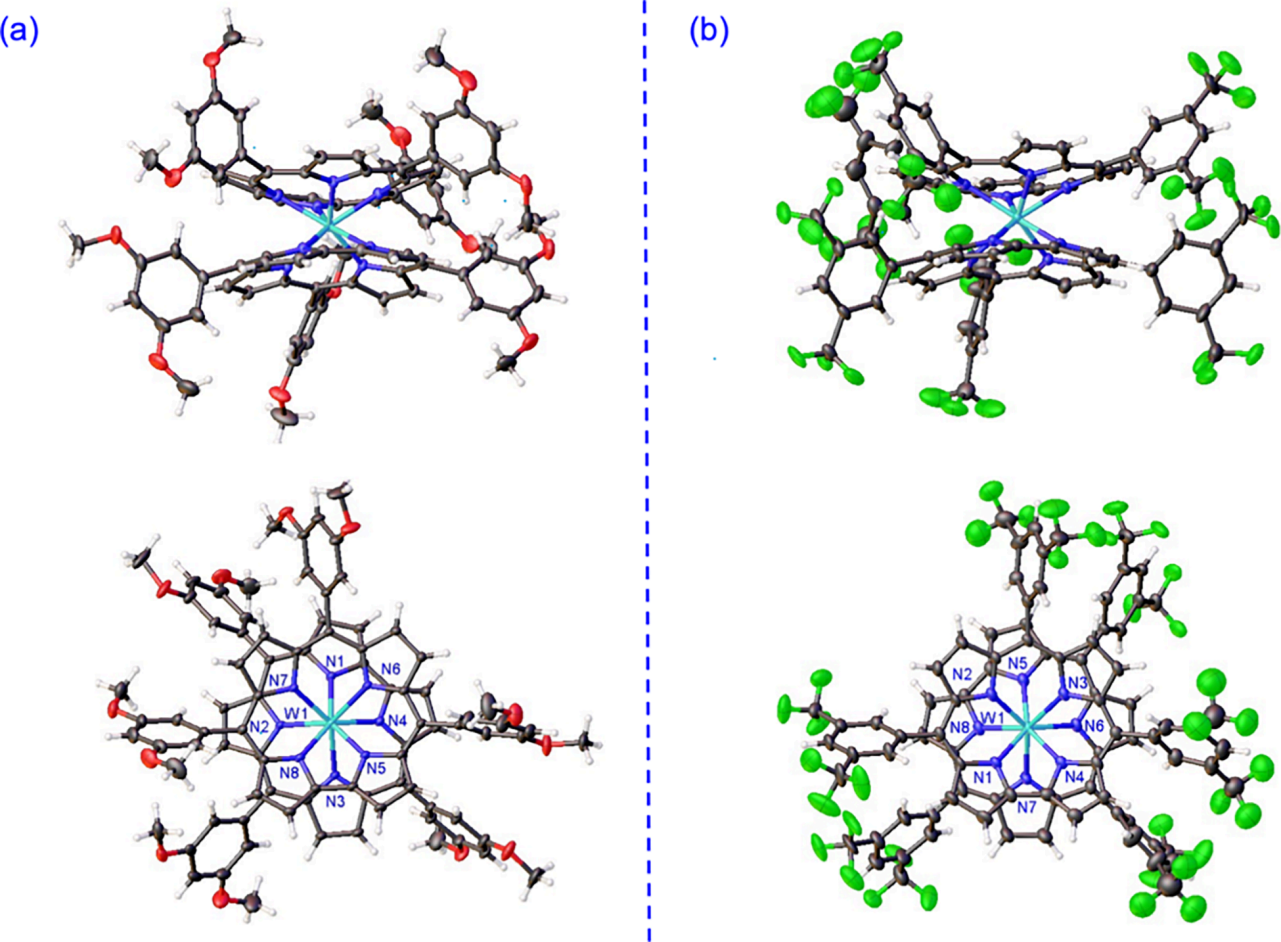
Thermal ellipsoid plots (side and top views) for (a) W[TDOMePC]_2_ (50% probability) and (b) W[TBCF_3_PC]_2_ (25% probability). Selected distances (Å) for W[TDOMePC]_2_: W1–N1 2.1467(12), W1–N2 2.2059(11), W1–N3 2.2126(12), W1–N4 2.1555(12), W1–N5 2.1512(11), W1–N6 2.2172(12), W1–N7 2.1942(11), and W1–N8 2.1449(12). Selected distances (Å) for W[TBCF_3_PC]_2_: W1–N1 2.162(6), W1–N2 2.201(7), W1–N3 2.207(7), W1–N4 2.161(7), W1–N5 2.148(7), W1–N6 2.219(6), W1–N7 2.219(7), and W1–N8 2.160(7).

The biscorrole complexes based on TDOMePC and TBCF_3_PC (but not TPFPC) were found to separate into two very closely spaced bands on preparative thin layer chromatography (PTLC) plates. Mass spectrometry indicated identical isotope patterns for both bands, but UV–vis analysis indicated slightly different peak positions, as expected for diastereomers with similar coordination geometries. Subsequently, ^1^H NMR spectroscopy also indicated similar but distinct spectra, again consistent with a diastereomeric relationship between the two bands. Attempts to obtain single-crystal X-ray structures only proved successful for the D135 diastereomers of W[TBCF_3_PC]_2_ and W[TDOMePC]_2_ (Figure 1 and Table 1), suggesting that the D45 diastereomers are substantially more difficult to crystallize. The crystal structures were found to be qualitatively quite similar to those of other metallobiscorroles reported earlier^11−13^ and, accordingly, do not warrant detailed discussion. The W–N distances vary within a 0.1-Å range, i.e., 2.18 ± 0.10 Å, reflecting subtle electronic heterogeneity of the corrole nitrogens. The D45 diastereomers, on the other hand, were assigned by elimination. Reassuringly, DFT (OLYP/ZORA-STO-TZ2P) calculations indicated the D45 and D135 conformations (and their enantiomers) as the only local minima along the potential energy surface related to the mutual rotation of the two corroles.

**Table 1:** Crystallographic Data for W[TDOMePC]_2_ and W[TBCF_3_PC]_2_

	W[TDOMePC]_2_	W[TBCF_3_PC]_2_
CCDC deposition no.	2385243	2420478
chemical formula	C_86_H_70_N_8_O_12_W	C_86_H_34_F_36_N_8_W
formula mass	1591.35	2047.06
crystal system	triclinic	triclinic
crystal size (mm^3^)	0.250 × 0.080 × 0.050	0.050 × 0.040 × 0.010
space group	*P*1̅	*P*1̅
λ (Å)	0.7288	0.7288
*a* (Å)	15.6781(12)	13.9669(15)
*b* (Å)	16.7951(13)	16.1457(17)
*c* (Å)	19.1372(14)	22.215(2)
α (deg)	104.384(3)	70.499(4)
β (deg)	105.823(3)	76.662(4)
γ (deg)	107.323(3)	80.166(4)
*Z*	2	2
*V* (Å^3^)	4320.0(6)	4570.8(9)
temperature (K)	100(2)	100(2)
density (g/cm^3^)	1.904	1.487
measured reflections	33098	13238
unique reflections	30045	11305
parameters	1016	1180
restraints	36	261
*R*_int_	0.0422	0.0807
θ range (deg)	1.395–34.217	1.379–24.019
*R*_1_, *wR*_2_ all data	0.0264, 0.0715	0.0766, 0.2308
*S* (GooF) all data	1.064	1.066
max/min res. dens. (e/Å^3^)	1.464/–1.317	2.246/–1.122

### ^1^H NMR Analysis

**Figure 2:**
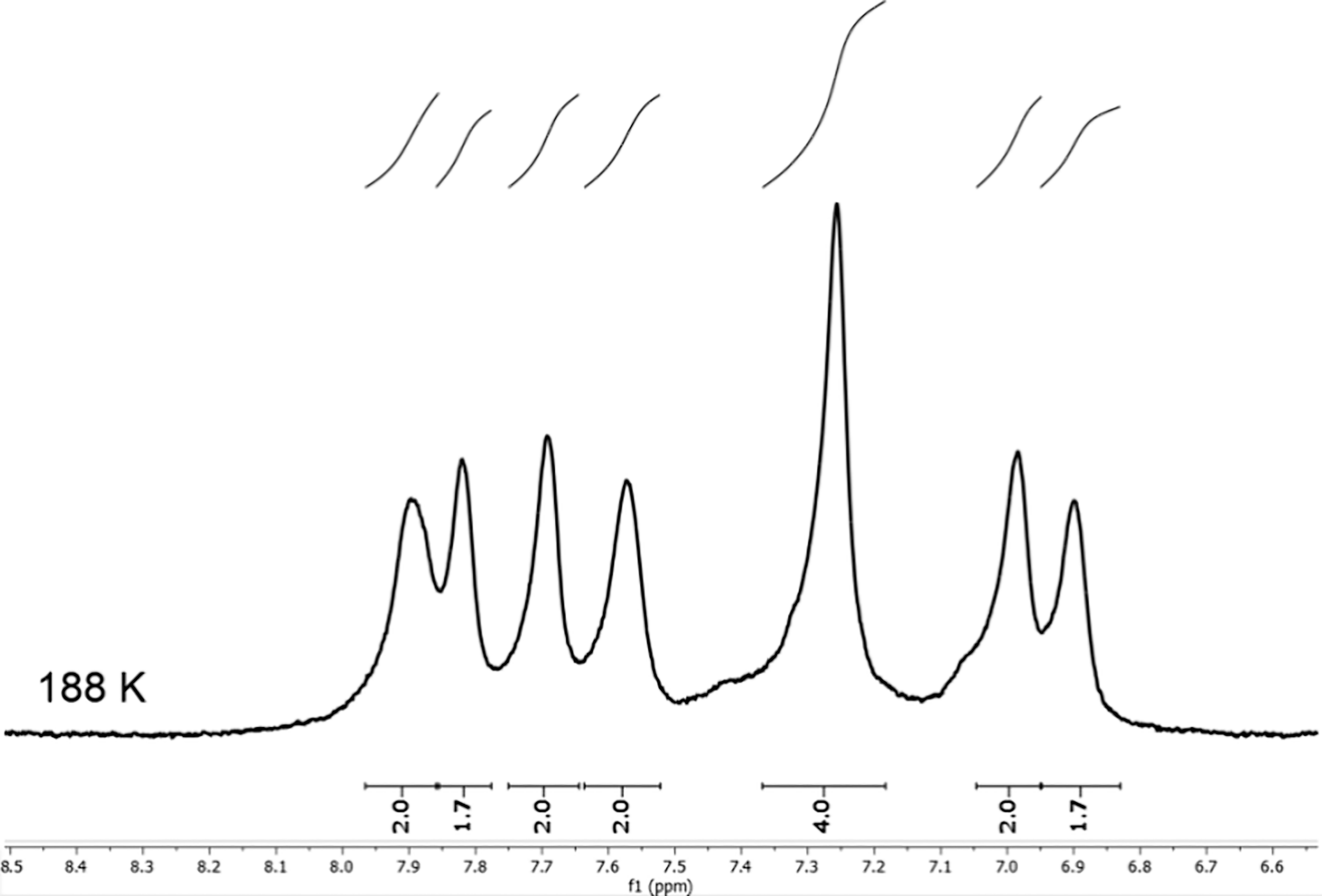
^1^H NMR spectrum of W[TPFPC]_2_ at 188 K in CD_2_Cl_2_.

Our choice of corrole ligands in this study was motivated by a desire to declutter the aromatic region of the ^1^H NMR spectra. The simplest spectra were expected for TPFPC complexes, which have no *meso*-aryl protons. Indeed, W[TPFPC]_2_ was found to exhibit the expected number β-H and *meso*-F signals, consistent with time-averaged *C*_*2*_ symmetry, under suitable conditions (Figure 2). Dynamic effects were observed for the *ortho*-F’s, indicating hindered rotation about the C*_meso_*-C_*ipso*(aryl)_ bonds.

**Figure 3:**
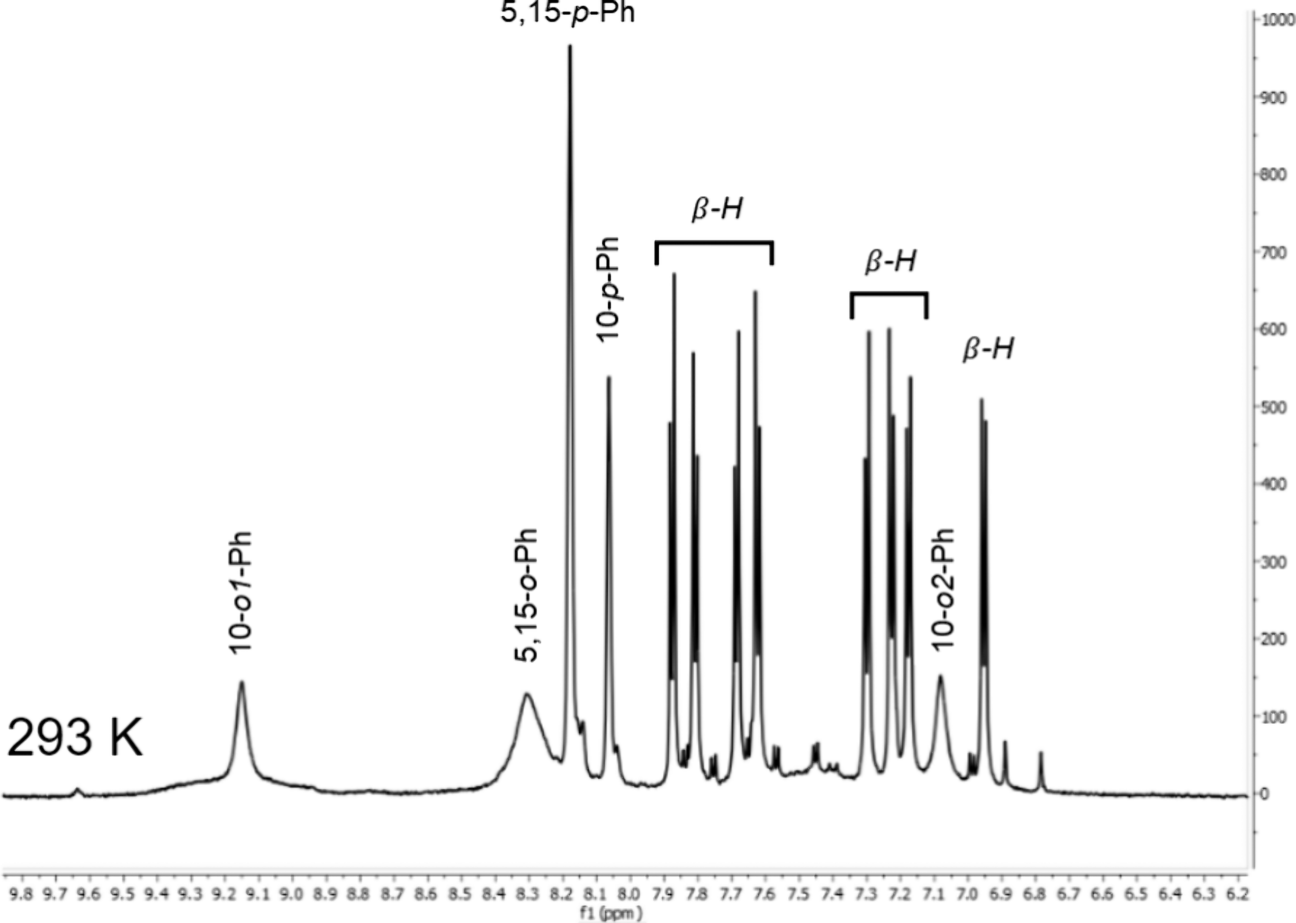
^1^H NMR spectrum of D135-W[TBCF_3_PC]_2_ at room temperature in CH_2_Cl_2_.

A high-quality ^1^H NMR spectrum was observed for D135-W[TBCF_3_PC]_2_ at room temperature (Figure 3). Full assignment of the spectrum was possible using a combination of the 1D and TOCSY spectra. The corrole β-protons were first assigned as the only *J*-coupled signals. These accounted for eight magnetic environments, each with an integral of two, i.e., a total of 16 protons. Next, the 5,15-*p*-aryl and 10-*p*-aryl protons were assigned on the basis of their integrals, 4 and 2, respectively. The remaining dynamically broadened peaks, accordingly, could be attributed to the 5,10,15-*o*-aryl protons. Fortunately, the ^1^H–^1^H TOCSY spectrum revealed coupling between the broad signals at 7.1 and 9.2 ppm and the 10-*p*-aryl protons (Figure S9). As a result, both the 7.1 and 9.2 ppm signals could be assigned as 10-*o*-aryl protons. By elimination, the broad signal at 8.3 ppm had to correspond to a coalescence of 5,15-*o*-aryl protons. As discussed below, these protons too could be fully resolved at 193 K. Overall, the ^1^H NMR spectrum of D135-W[TBCF_3_PC]_2_ was found to correspond to *C*_2_ symmetry on the NMR time scale.

The aforementioned dynamic effects exhibited by the *ortho* protons reflect restricted rotation around the C*_meso_*-C_*ipso*(aryl)_ bonds. For a given aryl group, the two *ortho* protons, when fully resolved, were found to exhibit a remarkably large chemical shift difference ∼ 2 ppm, indicating a dramatic difference in magnetic environment in the *endo* and *exo* regions around the molecule (these being the regions between and outside the two mean corrole planes, respectively). Given the strong aromaticity of the corrole macrocycle,^20,21^ the *endo* region is expected to be significantly less chemically shielded, leading to higher chemical shifts, relative to the *exo* region. Such magnetic anisotropy has also been noted for metalloporphyrin sandwich compounds. These dynamic effects may provide a partial explanation of the indecipherable ^1^H NMR spectra previously noted for Group 6 metallobiscorrole compounds.

**Figure 4:**
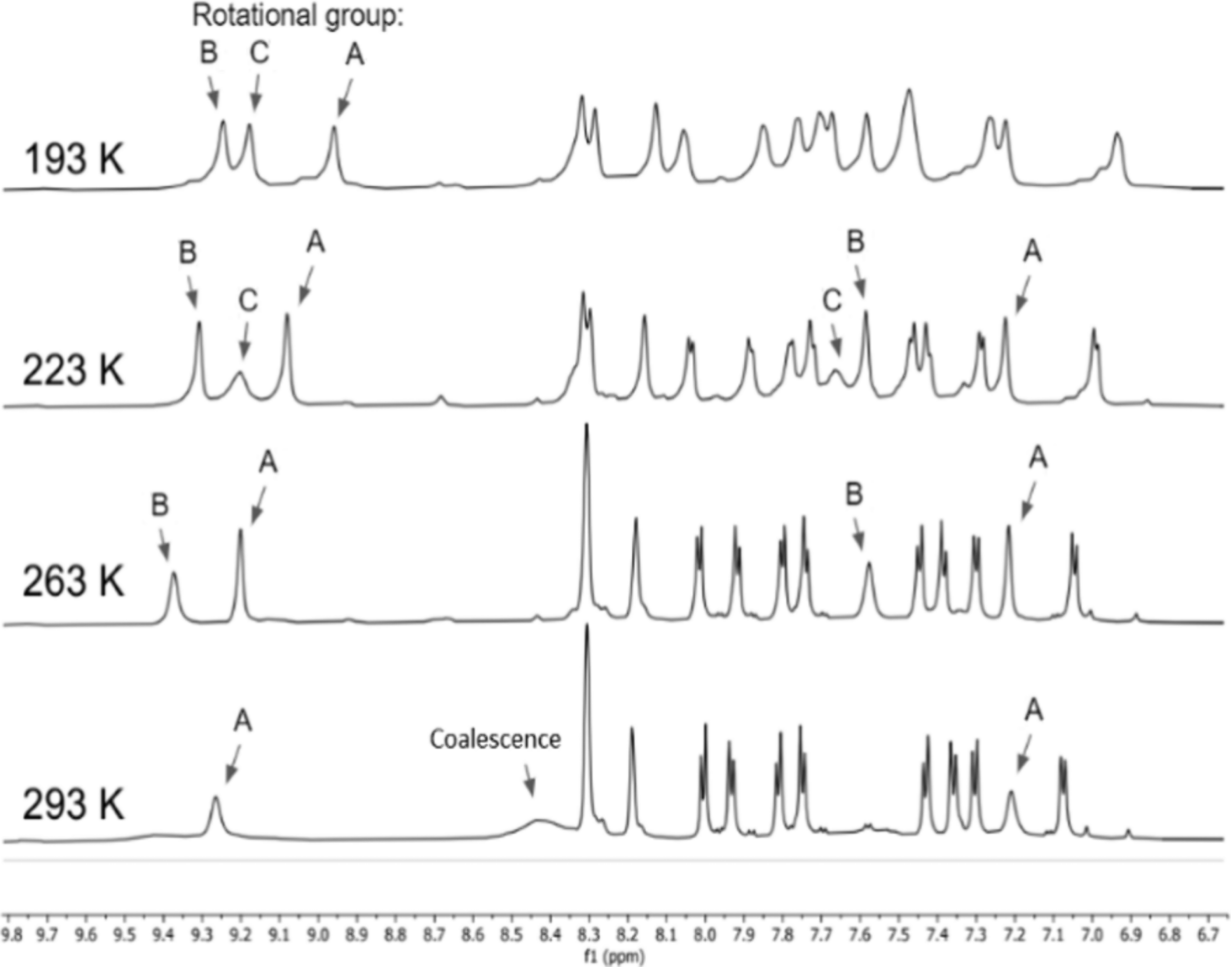
^1^H NMR spectra of W[TBCF_3_PC]_2_ D135 at 193, 223, 263, and 293 K (top to bottom) in CH_2_Cl_2_. The markers A, B, and C refer to *ortho* protons on the three symmetry-distinct *meso*-aryl groups. See Table 2 for selected dynamic parameters.

Variable-temperature ^1^H NMR experiments on D135-W[TBCF_3_PC]_2_ revealed details of the dynamic effects observed for the *o*-aryl positions (Figure 4, Figure S8 and Table 2). As the temperature was progressively lowered, the aryl rotations all slowed down to the point where all 6 symmetry-distinct *o*-aryl protons on each corrole appeared as distinct signals. The signals appeared as three *J*-coupled sets (denoted A, B and C), each consisting of the *endo* and *exo ortho* proton signals for a given aryl group, corresponding to the three symmetry-distinct aryl groups on each corrole macrocycle. It may be useful to note that the 5,15-aryl groups are not symmetry-related, as they are in many simple metallotriarylcorroles, because of the asymmetric local environment of each corrole (see ref 16 for a definition of the *local symmetry* of a part of molecule). Of these, set A, consisting of the 10-*o*-aryl protons, was already discernible as two separate signals at room temperature, indicating that this aryl group has the lowest rotational speed. The rotational barriers of all three symmetry-distinct aryl groups were evaluated with the Eyring equation and found to range over 11–14 kcal/mol. The range presumably reflects differences in both electronic character (5,15- versus 10-) and steric environment of the aryl groups.

**Table 2:** Rotational Barriers of *meso*-Aryl Groups A, B, and C in D135-W[TBCF_3_PC]_2_ (see Figure 4) Calculated Using Eyring’s Equation

rotational group	assignment	rotational barrier (kcal/mol)	coalescence frequency (Hz)	chemical shifts at start of slow-exchange regime (ppm)
A	10-*o*-Ph	14.1	1867	7.2, 9.3 (293 K)
B	5,15-*o*-Ph	13.3	1600	7.6, 9.4 (263 K)
C	5,15-*o*-Ph	11.1	1422	7.7, 9.3 (223 K)

**Figure 5:**
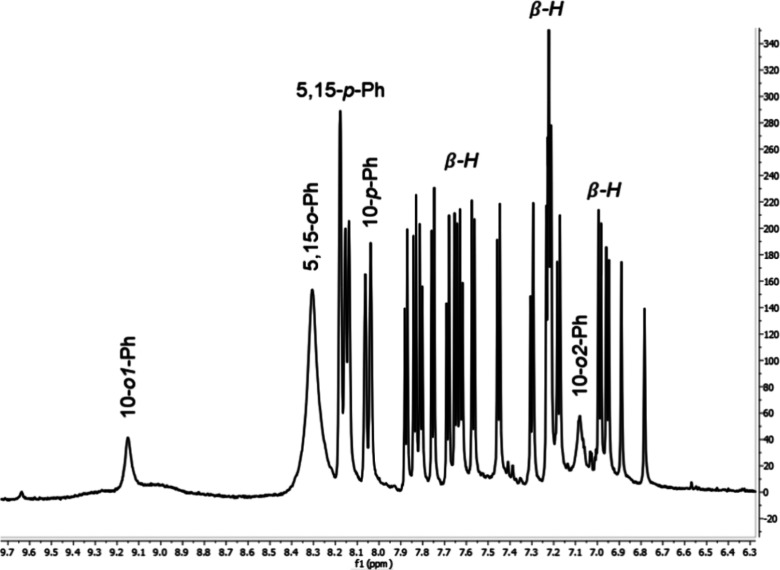
^1^H NMR spectrum of D45-W[TBCF_3_PC]_2_ at room temperature in CH_2_Cl_2_.

^1^H NMR analysis of the putative D45 diastereomer of W[TBCF_3_PC]_2_ proceeded much along the same lines as for the D135 isomer, except in one key respect: the D45 isomer does not exhibit *C*_2_ symmetry on the NMR time scale (Figure 5). The reasons for the loss of symmetry remain unclear for now; the fact that the D45 stereochemistry is significantly more sterically crowded may be a factor. Fortunately, the NMR spectrum was pretty much fully assignable, with the *o*-aryl protons exhibiting the same dynamic behavior as in the D135 diastereomer, with the slowest rotation observed for the 10-aryl groups.

The ^1^H NMR spectra of the two diastereomers of Mo[TBCF_3_PC]_2_ proved to be of lower quality in terms of both resolution and discernible *J*-coupling. We were able to arrive at a plausible distinction between the D45 and D135 diastereomers of Mo[TBCF_3_PC]_2_ based on (a) the similarity of peak positions relative to their tungsten counterparts and (b) dynamic effects observed for the *ortho* protons. Specifically, the putative D135 diastereomers were found to exhibit significantly slower 5,15-aryl rotations at room temperature relative to their D45 counterparts, as evident from a comparison of the integrals of the fast-equilibrating 5,15-*o*-aryl peaks at ∼ 8.3 ppm. Armed with that insight, we could assign the ^1^H NMR spectrum of the putative D135-Mo[TBCF_3_PC]_2_ diastereomer using the same arguments as those used for D135-W[TBCF_3_PC]_2_ structure; like its W counterpart, the Mo structure was found to conform to overall *C*_2_ symmetry. Dynamic effects confounded the full assignment of the room-temperature ^1^H NMR spectrum of D45-Mo[TBCF_3_PC]_2_, but at 213 K, the spectrum was largely assignable and consonant with *C*_2_ symmetry. Thus, among the four M[TBCF_3_PC]_2_ (M = Mo, W) species studied (including diastereomers), only D45-W[TBCF_3_PC]_2_ evinced a loss of *C*_2_ symmetry.

### Electronic Absorption Spectra and Electrochemistry

**Figure 6:**
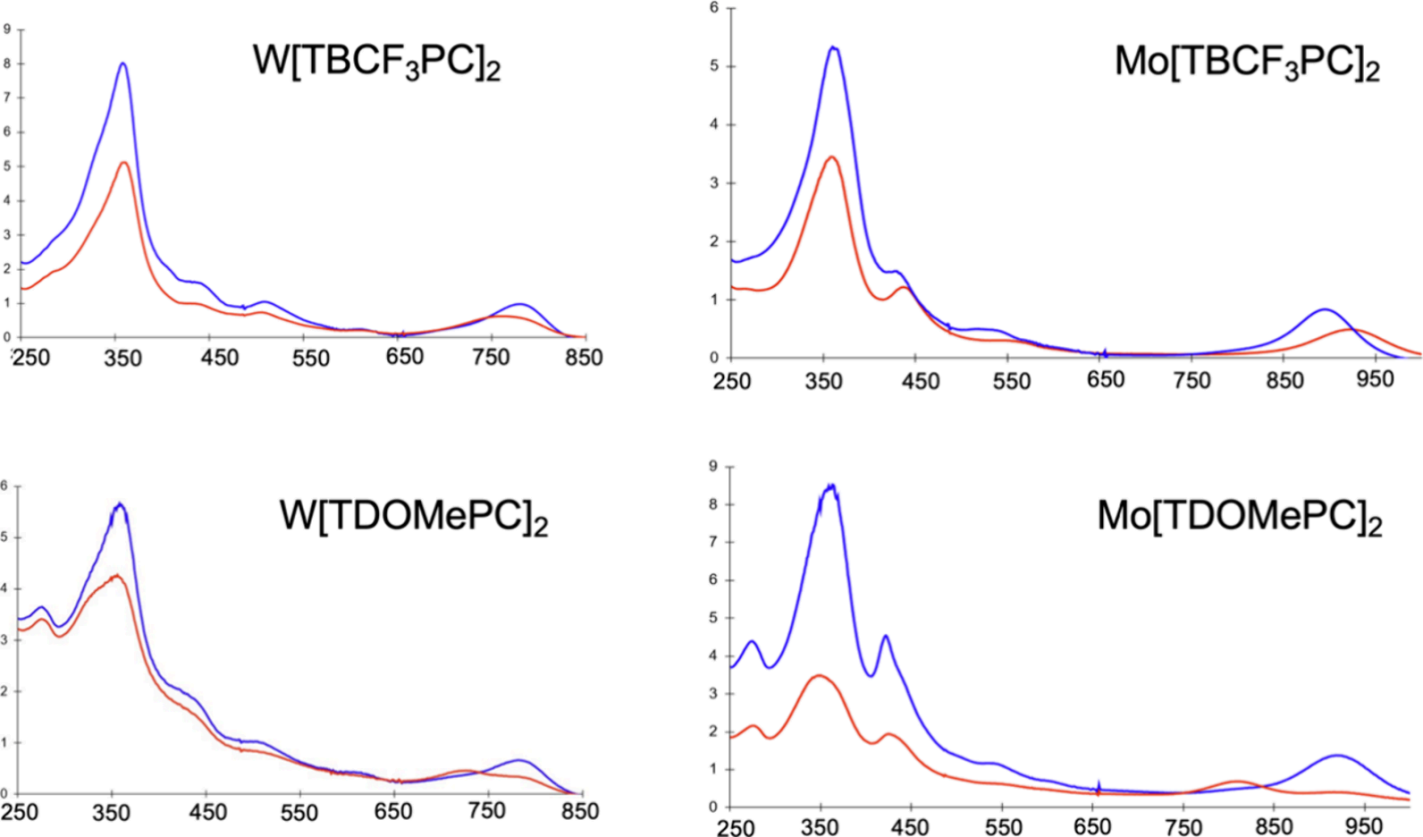
UV–vis spectra in dichloromethane at room temperature: ε × 10^–4^ (M^–1^cm^–1^) vs wavelength (nm). The faster- and slower-moving isomers, generally assigned as D135 and D45, respectively, are indicated in blue and red, respectively.

The new metallobiscorroles reported here exhibit UV–vis characteristics broadly similar to those previously reported for tungsten and molybdenum biscorroles, namely a blueshifted Soret band with λ_max_ ∼ 360 nm and a strongly redshifted Q-like band with λ_max_ ∼ 780 nm for tungsten and ∼ 900 nm for molybdenum (Figure 6). However, the diastereomers exhibit somewhat dissimilar Q-band profiles in terms of band shape and extinction coefficients. In the case of Mo[TDOMePC]_2_, the Q-band maxima of the two diastereomers differ rather substantially, by >100 nm. Earlier TDDFT studies attributed the Q-band absorption to a ligand-to-metal charge-transfer transition to an empty metal(d_z2_)-based LUMO. The difference in the absorption maxima may thus indicate a subtle difference in coordination geometry between the two diastereomers.

**Figure 7:**
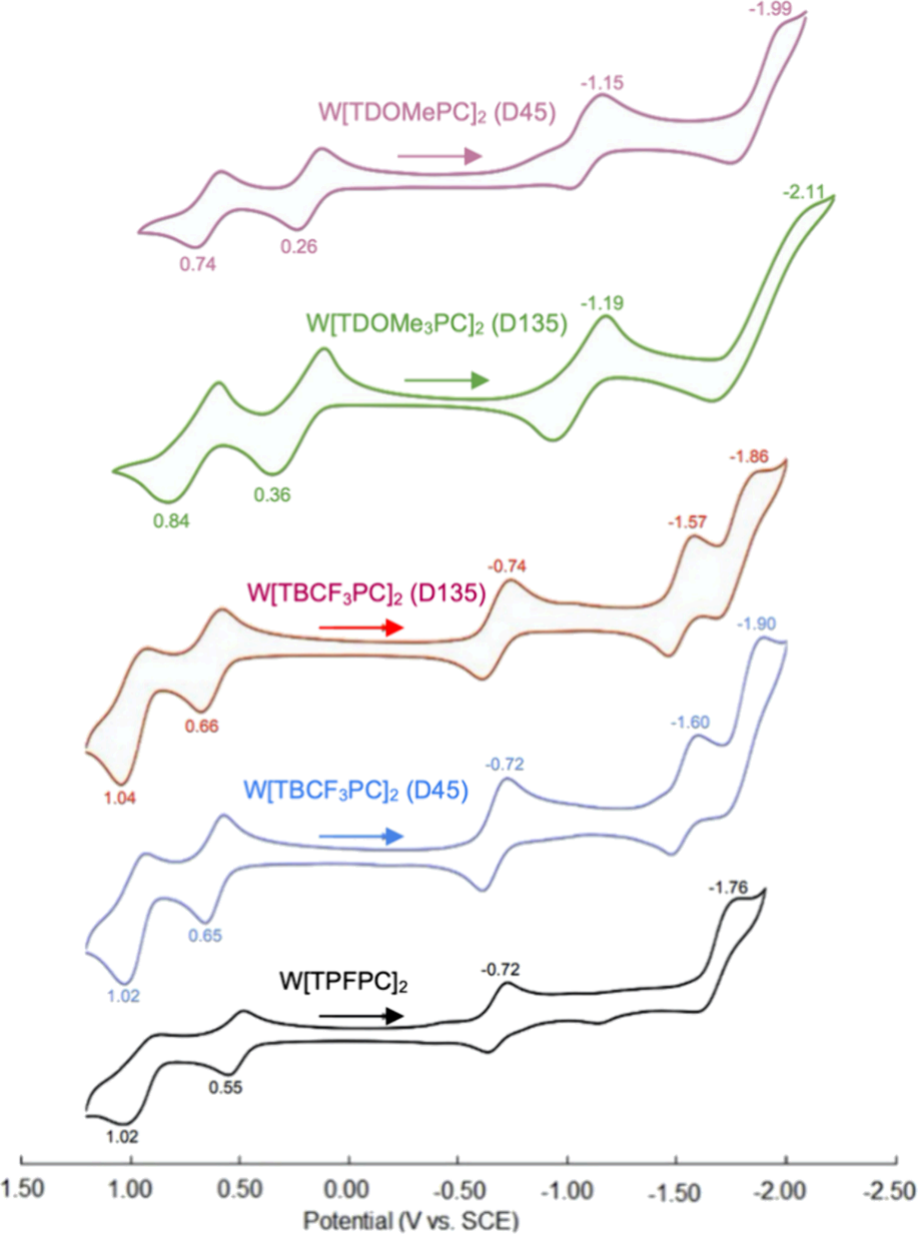
Cyclic voltammograms in dichloromethane with 0.1 M tetrabutylammonium perchlorate as the supporting electrolyte.

Unsurprisingly, the electrochemical behavior of the new tungsten biscorroles is broadly similar to that of those reported earlier, in terms of both redox potentials and the electrochemical HOMO–LUMO gap (Δ*E*, defined as the algebraic difference between the first oxidation and reduction potentials; Figure 7). For both W[TBCF_3_PC]_2_ and Mo[TDOMePC]_2_, the electrochemical HOMO–LUMO gaps exhibit the order D135 > D45, qualitatively consistent with the lowest-energy Q-band maxima of the two isomers (Table 3). Note also that the two diastereomers of W[TBCF_3_PC]_2_ exhibit a unique third reduction that is not observed for other metallobiscorroles, while the second reduction of W[TPFPC]_2_*may* correspond to a two-electron reduction, based on the height of the feature (Figure 7).

**Table 3:** Selected UV–vis Maxima (nm), Redox Potentials (V vs SCE), and Electrochemical HOMO–LUMO Gaps (V)

biscorrole	UV/vis		*E*_1/2red3_	*E*_1/2red2_	*E*_1/2red1_	*E*_1/2ox1_	*E*_1/2ox2_	Δ*E*
W[TPFPC]_2_	352	786	–1.76		–0.72	0.55	1.02	1.27
D135-W[T*m-*CF_3_PC]_2_	358	780	–1.90	–1.60	–0.72	0.65	1.02	1.37
D45-W[T*m-*CF_3_PC]_2_	361	755	–1.86	–1.57	–0.74	0.66	1.04	1.40
D135-W[TDOMe_3_PC]_2_	357	781	–1.99		–1.15	0.26	0.74	1.41
D45-W[TDOMe_3_PC]_2_	355	725	–2.11		–1.19	0.36	0.84	1.55

## Conclusions

Careful preparative thin-layer chromatography resulted in the separation of four metallobiscorroles, M[TBCF_3_PC]_2_ and M[TDOMePC]_2_ (M = Mo, W), into pairs of diastereomers. Such diastereomers were predicted to be configurationally stable on the basis of DFT calculations. The diastereomerism reflects the rigidity and robustness of the square-antiprismatic coordination in which the two corroles are rotated 45° or 135° relative to each other. For both tungsten complexes, the 135° diastereomer could be crystallographically analyzed, allowing the identification of the other diastereomer by elimination as the 45° diastereomer. Furthermore, each diastereomer is chiral and can exist as two enantiomers. The successful chromatographic resolution of the enantiomers has been documented earlier and has not been repeated in this study. The molybdenum complexes did not lend themselves to X-ray diffraction analysis so the identity of the stereoisomers remains somewhat speculative. That said, both isomers of W[TBCF_3_PC]_2_ and of Mo[TBCF_3_PC]_2_ could be comprehensively analyzed with ^1^H NMR spectroscopy, with the spectra qualitatively consistent with the proposed diastereomeric relationship. The metallobiscorrole framework thus emerges as doubly stereogenic, giving rise to both diastereomers and enantiomers – to our knowledge, a unique stereochemical scenario for sandwich compounds. Potential practical applications of these novel chiral materials remain a fascinating prospect for the future.

## Experimental Section

### Materials

Anhydrous decalin (mixture of isomers), tungsten and molybdenum hexacarbonyls (99.99%), and potassium carbonate (granulated) were purchased from Sigma-Aldrich and used as received. Silica gel 60 (0.04–0.063 mm particle size, 230–400 mesh, Merck) was employed for column chromatography. Silica gel 60 PTLC plates (20 × 20 cm; 0.5 mm thick, Merck) were used for the final purification of all complexes. The free-base corroles H_3_[TPFPC], H_3_[TDOMePC] and H_3_[TBCF_3_PC] were prepared according to literature procedures.^17−19^

### Instrumental Methods

UV–visible–NIR spectra were recorded on an HP 8453 spectrophotometer. ^1^H and ^19^F NMR spectra were recorded on a 400 MHz Bruker Avance III HD spectrometer equipped with a 5 mm SmartProbe in CD_2_Cl_2_ and referenced to residual CH_2_Cl_2_ at δ = 5.31 ppm. ^1^H NMR spectra were acquired between −85 and 25 °C, and ^19^F NMR spectra were acquired at room temperature. 2D-TOCSY NMR spectra were obtained using a mixing time of 80 μs. Electrospray ionization mass spectra were recorded on an LTQ Orbitrap XL spectrometer. Cyclic voltammetry was carried out in dry dichloromethane at 298 K with an EG&G Model 263 A potentiostat having a three-electrode system: a glassy carbon working electrode, a platinum wire counter-electrode and a saturated calomel reference electrode (SCE). Tetra(*n-*butyl)ammonium perchlorate, recrystallized twice from absolute ethanol and dried in a desiccator for at least 2 weeks, was used as the supporting electrolyte. Anhydrous CH_2_Cl_2_ (Aldrich) was used as the solvent. The electrolyte solution was sparged with argon for at least 5 min, and all measurements were carried out under an argon blanket. All potentials were referenced to the saturated calomel electrode.

#### Tungsten 5,10,15-Tris(pentafluorophenyl)biscorrole

Decalin (10 mL), H_3_[TPFPC] (52 mg, 0.065 mmol), W(CO)_6_ (137 mg, 0.39 mmol), and potassium carbonate (150 mg) were placed inside a 50 mL three-neck round-bottom flask equipped with a reflux condenser and a magnetic stirring bar. The stirred contents were deoxygenated with a flow of argon for 1 h and subsequently refluxed overnight with constant stirring under argon. Completion of the reaction was indicated by the appearance of a new Q-band absorption at ∼780 nm. Upon cooling, the reaction mixture was then loaded on to a silica gel column with *n*-heptane as the mobile phase. Decalin was first removed by eluting with pure *n*-heptane; the tungsten biscorrole was then eluted with 3:1 *n*-heptane/dichloromethane. All fractions with λ_max_ ∼ 780 nm were collected and dried. The resulting crude product was chromatographed again on a second silica gel column with 5:1 *n*-heptane/dichloromethane as eluent, and again all fractions with λ_max_ ∼ 780 nm were collected. The fractions were dried and the resulting product was subjected to PTLC with 2:1 *n*-heptane/dichloromethane as eluent. The tungsten biscorrole showed up as a distinct brown band on the PTLC plate, with no indication of multiple diastereomers. Yield 1.6 mg (2.78%). ^1^H NMR (400 MHz, CD_2_Cl_2_, −85 °C) δ 7.89 (m, 2H), 7.82 (m, 2H), 7.69 (m, 2H), 7.57 (m, 2H), 7.26 (m, 4H), 6.98 (m, 2H), 6.90 (m, 2H). ^19^F NMR (377 MHz, CD_2_Cl_2_) δ −128.70 – −132.04 (m, 4F, *o-*F), −137.10 (m, 4F, *o-*F), −153.07 – −153.85 (m, 6F, *p-*F), −161.86 – −162.67 (m, 12F, *m-*F). UV–vis (CH_2_Cl_2_) λ_max_ (nm) (ε×10^–4^, M^–1^ cm^–1^): 353 (5.22), 507 (0.66), 788 nm (0.82). HRMS (ESI): [M^+^] = 1770.0532 (expt), 1770.0535 (calcd for C_74_H_16_N_8_F_30_W).

#### Tungsten 5,10,15-Tris[3,5-bis(trifluoromethyl)phenyl]corrole

Decalin (15 mL), H_3_[TBCF_3_PC] (132.6 mg, 0.142 mmol), W(CO)_6_ (149.7 mg, 0.425 mmol), and potassium carbonate (100 mg) were placed inside a 100 mL two-neck round-bottom flask fitted with a reflux condenser and a magnetic stirring bar. The stirred mixture was deoxygenated with a flow of argon and then refluxed for 3 h (also under a flow of argon), at which point additional W(CO)_6_ (149.7 mg, 0.425 mmol) was added to the hot mixture. Refluxing was continued overnight with stirring under a constant flow of argon. Completion of the reaction was indicated by the appearance of a new Q-band in the 750–800 nm region. Upon cooling, the reaction mixture was loaded onto a silica gel column with *n*-heptane as the mobile phase. Decalin was first removed by eluting with pure *n*-heptane; the tungsten biscorrole was then eluted with 4:1 *n*-heptane/dichloromethane. All fractions with λ_max_ ∼ 770 nm were collected and dried. The resulting crude product was then chromatographed on a basic alumina column with 9:1 *n*-heptane/dichloromethane as eluent. Again, all fractions with λ_max_ ∼ 770 nm were collected and dried. The resulting product was purified by PTLC on a silica gel 60 plate with 3:1 *n*-heptane/dichloromethane as eluent, which led to splitting of the tungsten biscorrole into two isomers, which appeared as two reddish-brown bands. The faster-moving isomer (R_F_ = 0.40) exhibited a characteristic Q-band with λ_max_ ∼ 780 nm, while the slower-moving isomer (R_F_ = 0.29) exhibited a somewhat blueshifted Q-band with λ_max_ ∼ 750 nm. (*Note: Hereafter and in the SI, the descriptions faster- and slower-moving refer to movement of the species in question on a PTLC plate.*) W[TBCF_3_PC]_2_ was obtained as a brown solid with a total yield of 61.5 mg (42.4%) with a composition of 58.2 mg (40.1%) of isomer 1 and 3.3 mg (2.3%) of isomer 2.

#### W[TBCF_3_PC]_2_ (Faster-Moving Isomer, Later Identified as D135)

Yield 58.2 mg (40.10%). UV–vis (CH_2_Cl_2_) λ_max_ (nm) (ε×10^–4^, M^–1^ cm^–1^): 359 (12.25), 780 nm (1.87 M^–1^ cm^–1^). ^1^H NMR (400 MHz, CD_2_Cl_2_) δ 9.40 (s, 1H, 5- or 15-*o*), 9.26 (s, 2H, 10-*o*), 8.42 (s, 3H, 5,15-o), 8.31 (s, 4H, 5,15-p), 8.19 (s, 2H, 10-p), 8.00 (d, *J* = 4.6 Hz, 2H, β-H), 7.93 (d, *J* = 4.6 Hz, 2H, β-H), 7.81 (d, *J* = 4.6 Hz, 2H, β-H), 7.75 (d, *J* = 4.6 Hz, 2H, β-H), 7.43 (d, *J* = 4.9 Hz, 2H, β-H), 7.35 (d, *J* = 4.8 Hz, 2H, β-H), 7.30 (d, *J* = 4.8 Hz, 2H, β-H), 7.21 (s, 2H, 10-*o*), 7.08 (d, *J* = 4.7 Hz, 2H, β-H). ^19^F NMR (377 MHz, CDCl_3_) δ −62.19 – −63.42 (m). HRMS (ESI): [M^+^] = 2046.1847 (expt), 2046.1849 (calcd for C_86_H_34_F_36_N_8_W).

#### W[TBCF_3_PC]_2_ (Slower-Moving Isomer)

Yield 3.3 mg (2.27%). UV–vis (CH_2_Cl_2_) λ_max_ (nm) (ε×10^–4^, M^–1^ cm^–1^): 361 (7.54), 750 nm (1.01 M^–1^ cm^–1^). ^1^H NMR (400 MHz, CD_2_Cl_2_) δ 9.28 (s, 1H, 10-*o*), 8.43 (s, 6H, 5,10,15-*o*), 8.33 – 8.25 (m, 4H, 5,15-*p*), 8.19 (s, 1H, 10-*p*), 8.17 (s, 1H, 10-*o*), 8.01 (d, *J* = 4.6 Hz, 1H, β-H), 7.97 (d, *J* = 4.6 Hz, 1H, β-H), 7.94 (d, *J* = 4.6 Hz, 1H, β-H), 7.88 (d, *J* = 4.7 Hz, 1H, β-H), 7.81 (d, *J* = 4.6 Hz, 1H, β-H), 7.78 (d, *J* = 4.6 Hz, 1H, β-H), 7.75 (d, *J* = 4.6 Hz, 1H, β-H), 7.70 (d, *J* = 4.7 Hz, 1H, β-H), 7.58 (d, *J* = 4.7 Hz, 1H, β-H), 7.43 (d, *J* = 4.9 Hz, 1H, β-H), 7.38 – 7.33 (m, 3H, β-H), 7.31 (d, *J* = 4.8 Hz, 1H, β-H), 7.21 (s, 1H, 10-*o*), 7.12 (d, *J* = 4.7 Hz, 1H, β-H), 7.08 (d, *J* = 4.7 Hz, 1H, β-H). ^19^F NMR (377 MHz, CDCl_3_) δ −62.18 – −63.42 (m). HRMS (ESI): [M^+^] = 2047.1925 (expt), 2046.1849 (calcd for C_86_H_34_F_36_N_8_W).

#### Tungsten 5,10,15-Tris(3,5-dimethoxyphenyl)corrole

Decalin (15 mL), H_3_[TDOMePC] (100.0 mg, 0.141 mmol), W(CO)_6_ (149.7 mg, 0.425 mmol), and potassium carbonate (100 mg) were placed inside a 100 mL two-neck round-bottom flask fitted with a reflux condenser and a magnetic stirring bar. The stirred mixture was deoxygenated with a flow of argon and then refluxed for 3 h (also under a flow of argon), at which point additional W(CO)_6_ (149.7 mg, 0.425 mmol) was added to the hot mixture. Refluxing was continued overnight with stirring under a constant flow of argon. Completion of the reaction was indicated by the appearance of a new Q-band in the 750–800 nm region. Upon cooling, the reaction mixture was loaded directly onto a silica gel column with *n*-heptane as the mobile phase. The decalin was first removed by eluting with pure *n*-heptane; the tungsten biscorrole was then gradient-eluted, starting with dichloromethane and progressing to 3:1 dichloromethane/ethyl acetate. All fractions with λ_max_ ∼ 750 nm were collected and dried. The resulting crude product was then chromatographed on a basic alumina column with 2:1 heptane/ethyl acetate as the eluent, followed by two consecutive PTLC runs on silica gel 60 plates (20 cm × 20 cm) with 1:1 heptane/ethyl acetate as the eluent. The latter led to the splitting of the tungsten biscorrole into two isomers. The faster-moving isomer appeared as a brown band with a characteristic Q-band at 750 nm, while the slower isomer remained at the baseline, with a blueshifted Q-band with λ_max_ ∼ 700 nm.th

#### W[TDOMePC]_2_ (Faster-Moving Isomer, Later Identified as D135)

Yield 6.7 mg (5.97%). UV–vis (CH_2_Cl_2_) λ_max_ (nm) (ε×10^–4^, M^–1^ cm^–1^): 277 (7.62), 360 (17.39), 433 (4.32), 505 (2.64), 785 nm (2.82 m^–1^ cm^–1^). HRMS (ESI): [M^+^] = 1590.4630 (expt), 1590,4623 (calcd for C_86_H_70_O_12_N_8_W).

#### W[TDOMePC]_2_ (Slower-Moving Isomer)

Yield 5.0 mg (4.46%). UV–vis (CH_2_Cl_2_) λ_max_ (nm) (ε×10^–4^, M^–1^ cm^–1^): 277 (3.15), 341 (4.66), 427 (1.98), 724 nm (1.28). HRMS (ESI): [M^+^] = 1590.4634 (expt), 1590.4623 (calcd for C_86_H_70_O_12_N_8_W).

#### Molybdenum 5,10,15-Tris[3,5-bis(trifluoromethyl)phenyl]biscorrole

Decalin (10 mL), H_3_[TBCF_3_PC] (39.6 mg, 0.043 mmol), Mo(CO)_6_ (70 mg, 0.265 mmol), and potassium carbonate (150 mg) were placed inside a 50 mL three-neck round-bottom flask equipped with a reflux condenser and a magnetic stirring bar. The stirred contents were deoxygenated with a flow of argon and then refluxed overnight with constant stirring under argon. Completion of the reaction was indicated by the appearance of a new Q-band absorption with λ_max_ ∼ 900 nm. Upon cooling, the reaction mixture was loaded directly on to a silica gel column with *n*-heptane as the mobile phase. The decalin was first removed by eluting with pure *n*-heptane, and the molybdenum biscorrole was then eluted with 6:2:1 *n*-heptane/dichloromethane/acetone. All fractions with λ_max_ ∼ 900 nm were collected and evaporated to dryness. The crude product was then subjected to two successive PTLC runs on silica gel 60 plates, the first with 4:1 *n*-heptane/dichloromethane as eluent, and the second with 10:2:1 *n*-heptane/dichloromethane/acetone as eluent. The molybdenum biscorrole appeared as a broad brown band on the final TLC plate, whose leading and trailing edges gave distinct UV–vis spectra, consistent with two stereoisomers.

#### Mo[TBCF_3_PC]_2_ (Faster-Moving Isomer)

Yield 9.6 mg (6.77%). UV–vis (CH_2_Cl_2_) λ_max_ (nm) (ε×10^–4^, M^–1^cm^–1^): 359 (3.45), 437 (1.22), 923 (0.49). ^1^H NMR (400 MHz, CD_2_Cl_2_) δ 8.52 (s, 2H, 10-*o*), 8.13 (s, 4H, 5,15-*o*), 8.06 (s, 4H, 5- and 15-*p*), 7.93 (s, 2H, 10-*p*), 7.47 (d, *J* = 4.7 Hz, 2H, β-H), 7.30 (s, 2H, β-H), 7.23 (s, 2H, 10-*o*), 7.15 (s, 4H, β-H), 6.57 (s, 6H, β-H), 6.33 (s, 2H, β-H). HRMS (ESI): [M^+^] = 1961.1469 (expt), 1961.1458 (calcd for C_86_H_34_F_36_N_8_Mo).

#### Mo[TBCF_3_PC]_2_ (Slower-Moving Isomer)

Yield 5.2 mg (3.66%). UV–vis (CH_2_Cl_2_) λ_max_ (nm) (ε×10^–4^, M^–1^ cm^–1^): 360 (5.31),430 (1.49), 895 (0.84). ^1^H NMR (400 MHz, CD_2_Cl_2_) δ 8.43 (s, 3H), 8.17 (s, 4H, 5,15-*o*), 8.11 (s, 4H, 5,15-*o*), 8.06 (s, 2H, 5- or 15-*p*), 8.02 (s, 2H, 5- or 15-*p*), 7.95 (s, 2H, 10-*p*), 7.31 (m), 7.15 (m), 6.95 – 6.41 (m). HRMS (ESI): [M^+^] = 1961.1475 (expt), 1961.1458 (calcd for C_86_H_34_F_36_N_8_Mo).

#### Molybdenum 5,10,15-Tris(3,5-dimethoxyphenyl)corrole

Decalin (15 mL), H_3_[TDOMePC] (100 mg, 0.141 mmol), Mo(CO)_6_ (112 mg, 0.425 mmol) and potassium carbonate (100 mg) were added to a 50 mL two-neck round-bottom flask equipped with a reflux condenser and a magnetic stirring bar. The contents were deoxygenated with argon and then refluxed for 3 h, at which point additional Mo(CO)_6_ (112.2 mg, 0.425 mmol) was added to the hot mixture. Refluxing was continued overnight with constant stirring under argon. Completion of the reaction was indicated by the appearance of a new Q-band >800 nm. Upon cooling, the reaction mixture was loaded directly onto a silica gel column with *n*-heptane as the mobile phase. The decalin was first removed by eluting with pure *n*-heptane, and the molybdenum biscorrole was then eluted with 4:1 *n*-heptane/ethyl acetate progressing to 2:1 *n*-heptane/ethyl acetate. All fractions with a Q-band λ_max_ > 800 nm were collected and evaporated to dryness. The resulting crude product was purified by two consecutive PTLC runs on silica gel 60 plates with 1:1 *n*-heptane/ethyl acetate as the eluent. The second run led to the splitting of the molybdenum biscorrole into two isomers. The faster-moving diastereomer (*R*_F_ = 0.47) appeared as a brown band with a Q-band λ_max_ at 918 nm, while the other diastereomers remained at the baseline (*R*_F_ = 0), with a blueshifted Q-band λ_max_ at 809 nm.

#### Mo[TDOMePC]_2_ (Faster-Moving Isomer)

Yield 3.8 mg (3.6%). UV–vis (CH_2_Cl_2_) λ_max_ (nm) (ε×10^–4^, M^–1^ cm^–1^): 275 (4.39), 359 (8.49), 422 (4.54), 918 nm (1.38 m^–1^ cm^–1^). HRMS (ESI): [M^+^] = 1504.4209 (expt), 1504,4187 (calcd for C_86_H_70_O_12_N_8_Mo).

#### Mo[TDOMePC]_2_ (Slower-Moving Isomer)

Yield 0.6 mg (0.6%). UV–vis (CH_2_Cl_2_) λ_max_ (nm) (ε×10^–4^, M^–1^ cm^–1^): 276 (2.16), 348 (3.49), 425 (1.95), 811 nm (0.69). HRMS (ESI): [M^+^] = 1504.4192 (expt), 1504.4187 (calcd for C_86_H_70_O_12_N_8_Mo).

#### Single-Crystal X-ray Diffraction Analysis

Crystals suitable for single-crystal X-ray diffraction analysis were obtained via slow diffusion of heptane into concentrated solutions of the complexes in dichloromethane or chloroform. X-ray data were collected on beamline 12.2.1 at the Advanced Light Source, Lawrence Berkeley National Laboratory. Samples were mounted on MiTeGen Kapton loops and placed in a 100(2)-K nitrogen cold stream provided by an Oxford Cryostream 800 Plus low-temperature apparatus on the goniometer head of a Bruker D8 diffractometer equipped with a PHOTON II CPAD detector operating in shutterless mode. Diffraction data were collected using synchrotron radiation monochromated using Si(111) to a wavelength of 0.7288(1) Å. An approximate full-sphere of data was collected using a combination of φ and ω scans with scan speeds of one second per degree. The structures were solved by intrinsic phasing (SHELXT^22^) and refined by full-matrix least-squares on F^2^ (SHELXL-2014^23^). All non-hydrogen atoms were refined anisotropically. Hydrogen atoms were geometrically calculated and refined as riding atoms. For W[TDOMePC]_2_, a solvent mask was applied within the Olex2^24^ refinement software to highly disordered or ill-defined solvent molecules in the crystal.

As the reflection intensity decreased significantly at higher resolution for W[TBCF_3_PC]_2_, the data were truncated to a resolution of 0.90 Å, as this was determined to be the highest resolution shell with an *R*_int_ lower than 25%. To ensure correct behavior of part of the molecule (in particular the CF_3_ groups), the SADI and RIGU restraints were employed, along with the ISOR and DFIX constraints. Lastly, a solvent mask was applied within Olex2 to remove undetermined and poorly resolved solvent molecules. This structure is considered adequate for proof of molecular shape, stereochemistry, packing, etc., but bond distances and angles are likely to involve significant errors.

#### Density Functional Theory Calculations

DFT calculations were carried out in the gas phase under *C*_*2*_ symmetry with the scalar relativistic ZORA (Zeroth Order Regular Approximation to the Dirac equation) Hamiltonian,^25^ the OLYP^26,27^ exchange-correlation functional, Grimme’s D3 dispersion correction,^28,29^ ZORA STO-TZ2P all-electron basis sets, and a *C*_2_ symmetry constraint, all as implemented in the ADF 2019 program system.^30^ Carefully tested, fine grids and strict convergence criteria were used throughout.

## Data Availability

All data generated or analyzed in this study are included in this published article and its Supporting Information.

## Supporting Information Available

Additional spectroscopic results and optimized DFT coordinates (PDF)
