# Supplementary material for: Doubly Stereogenic Sandwich Frameworks: Diastereomeric Metallobiscorroles
Source: Inorg Chem. 2025 May 5;64(19):9621–30. doi: 10.1021/acs.inorgchem.5c00598 (PMC12093380; doi:10.1021/acs.inorgchem.5c00598)
Supplement: Supplementary file 1 — ic5c00598_si_001.pdf [file ic5c00598_si_001.pdf]

Supporting Information

Doubly Stereogenic Sandwich Frameworks: Diastereomeric Metallobiscorroles

Kristian Torstensen,[a] Florian Sixt,[a] Abraham B. Alemayehu,[a] Nicholas S. Settineri,[b] and Abhik Ghosh*,[a]

Address correspondence to: abhik.ghosh@uit.no (AG)

[a]Department of Chemistry, University of Tromsø, N-9037 Tromsø, Norway

[b]Advanced Light Source, Lawrence Berkeley National Laboratory, Berkeley, CA 94720-8229, United States.

Contents S1
$W[TPFPC]_2$ S2
$W[TBCF_3PC]_2$, D135 S5
$W[TBCF_3PC]_2$, D45 S8
$W[TDOMePC]_2$ S11
$Mo[TBCF_3PC]_2$, isomer 1 S13
$Mo[TBCF_3PC]_2$, isomer 2 S17
DFT optimized coordinates S20

W[TPFPC]$_2$

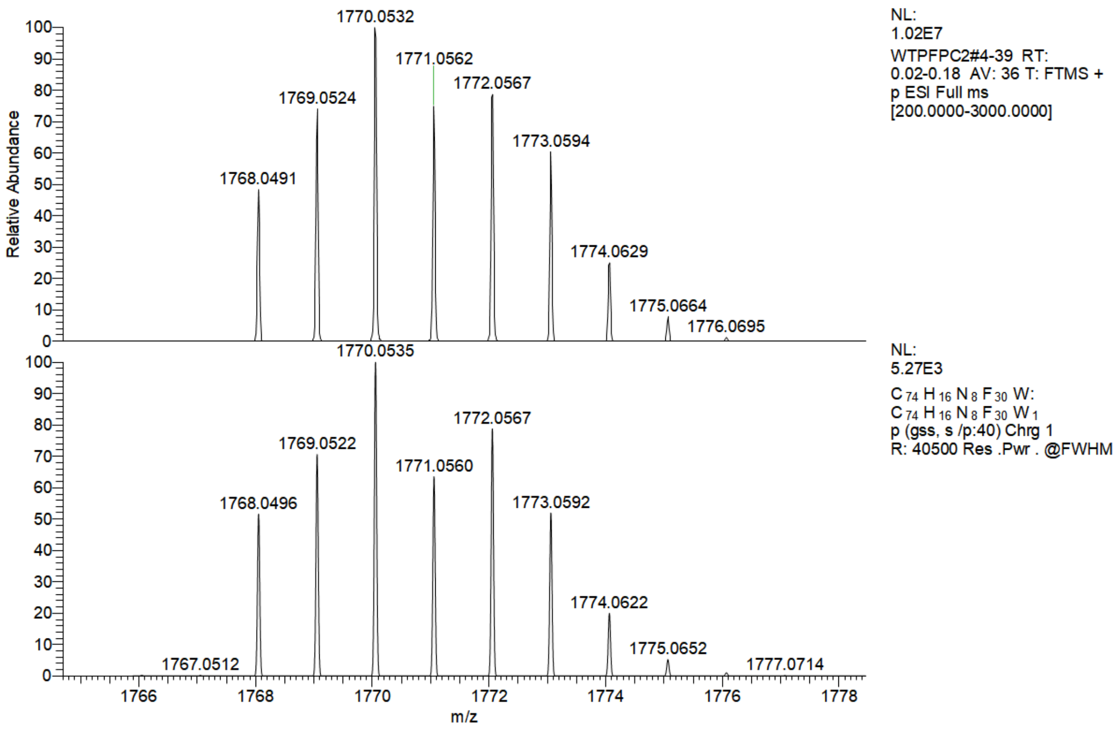

Figure S1. HRMS (top) of W[TPFPC]$_2$ with theoretical simulation (bottom).

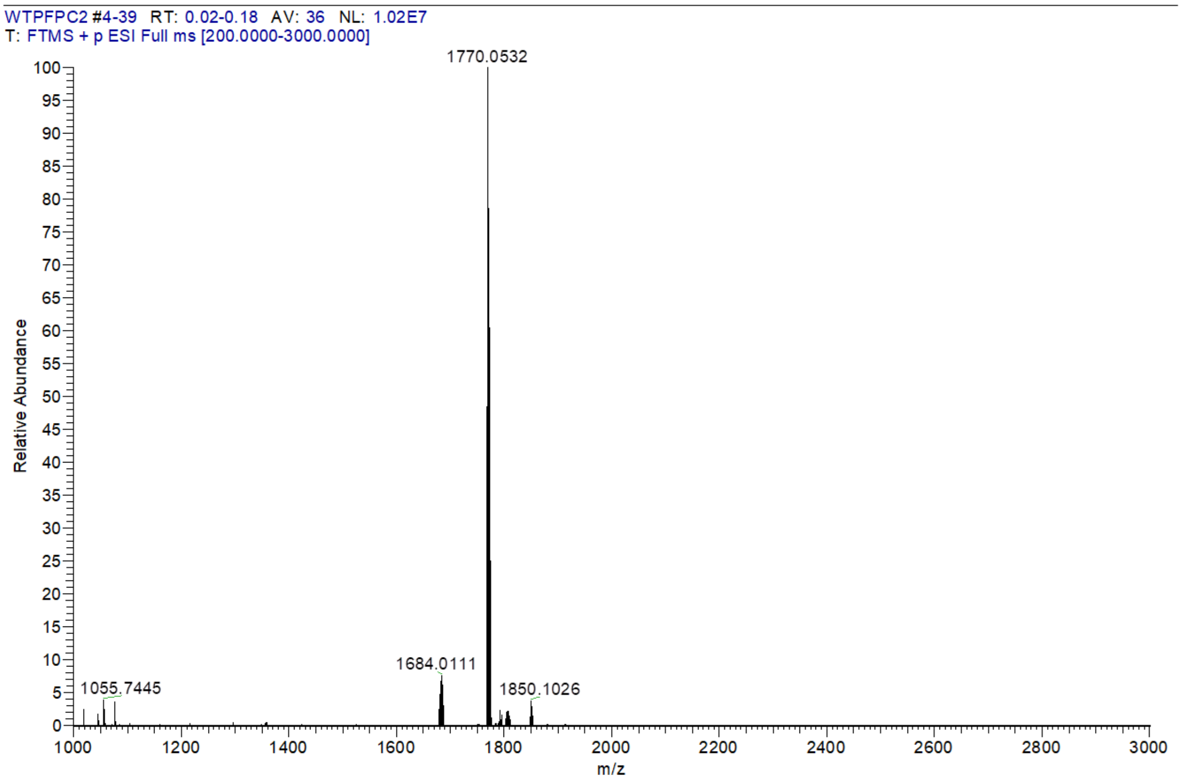

Figure S2. HRMS of W[TPFPC]$_2$.

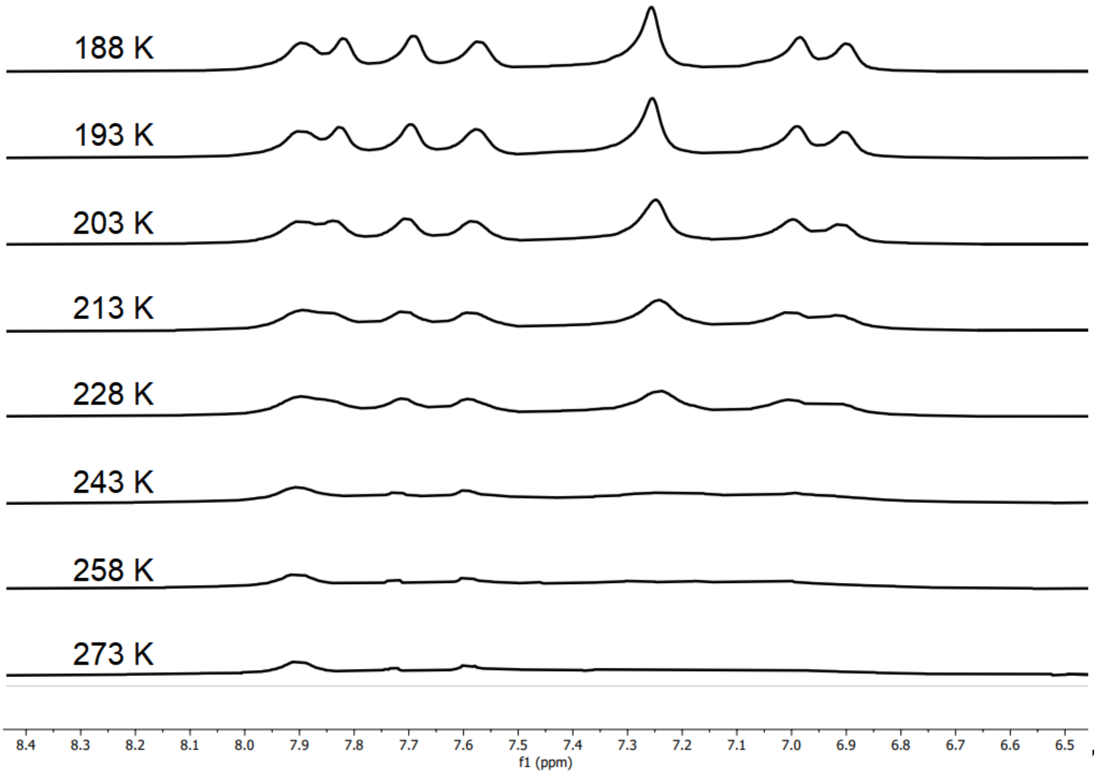

Figure S3. Variable-temperature ^{1}H NMR spectra of W[TPFPC]$_2$ in dichloromethane-d_2.

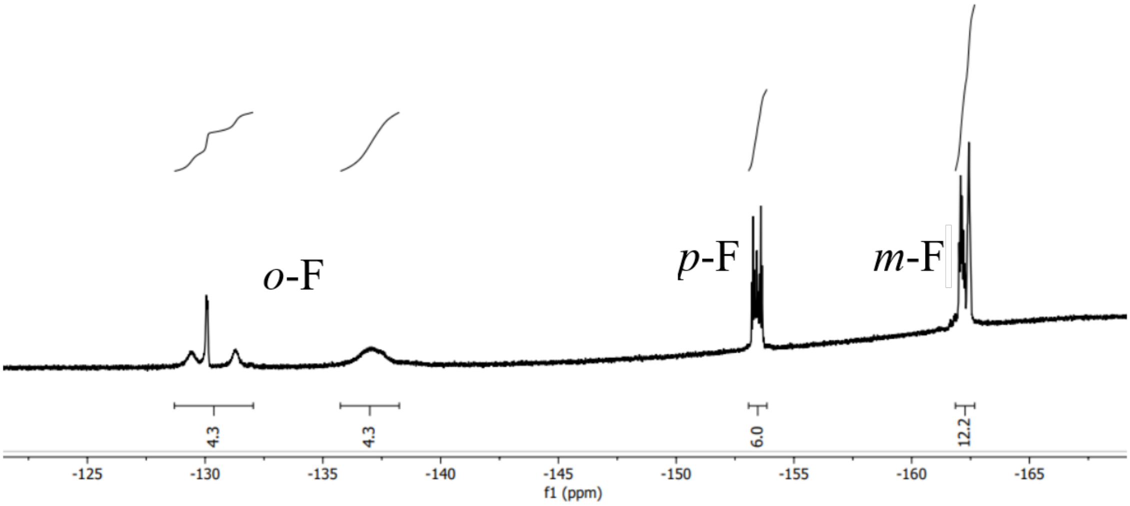

Figure S4. ^{19}F NMR spectrum of W[TPFPC]$_2$ in dichloromethane-d_2 at room temperature.

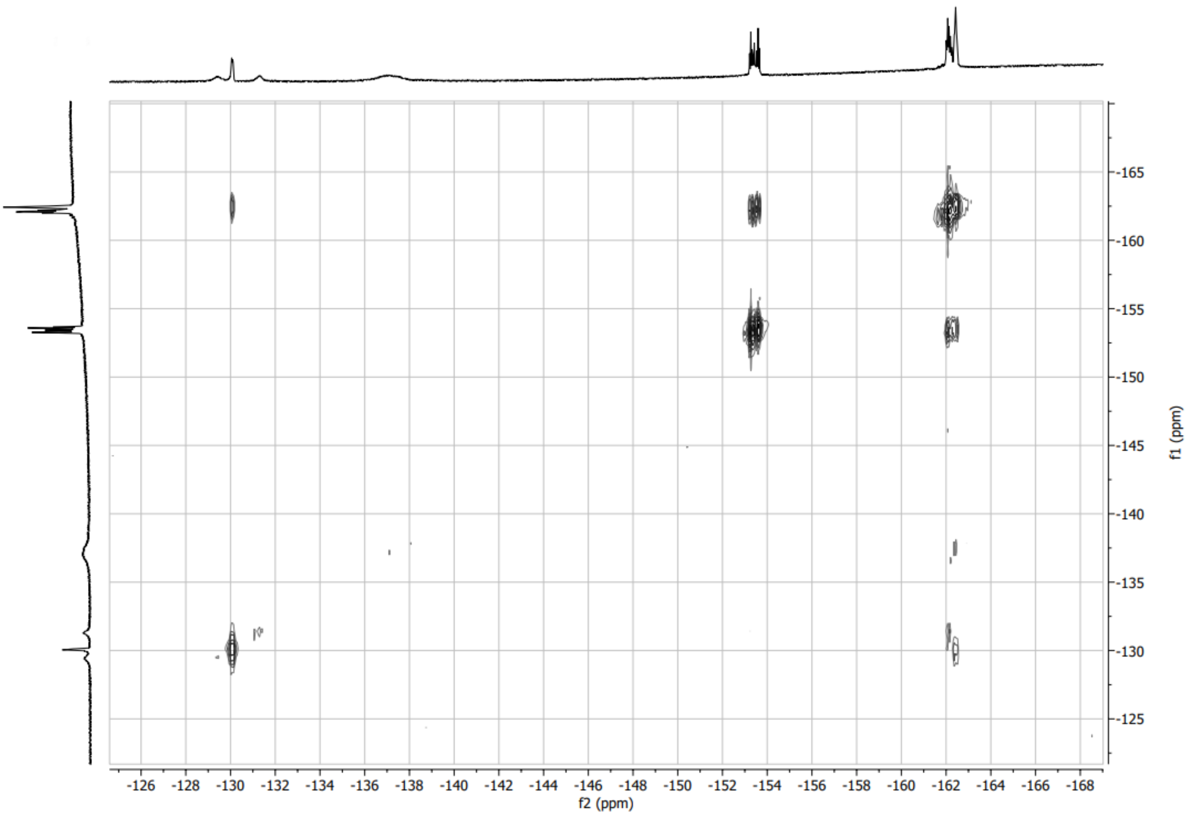

Figure S5. ^{19}F-^{19}F COSY of W[TPFPC]$_2$ in dichloromethane-d_2 at room temperature.

D135-W[TBCF$_3$PC]$_2$

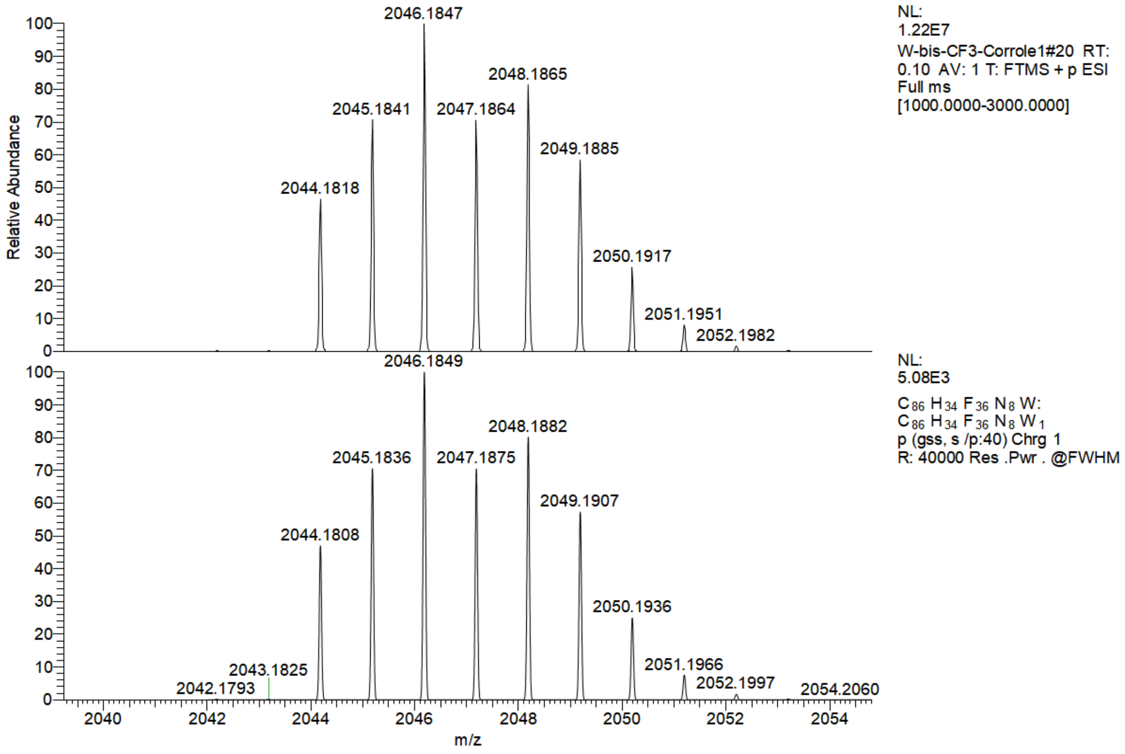

Figure S6. HRMS of D135-W[TBCF$_3$PC]$_2$ (top) and theoretical simulation (bottom).

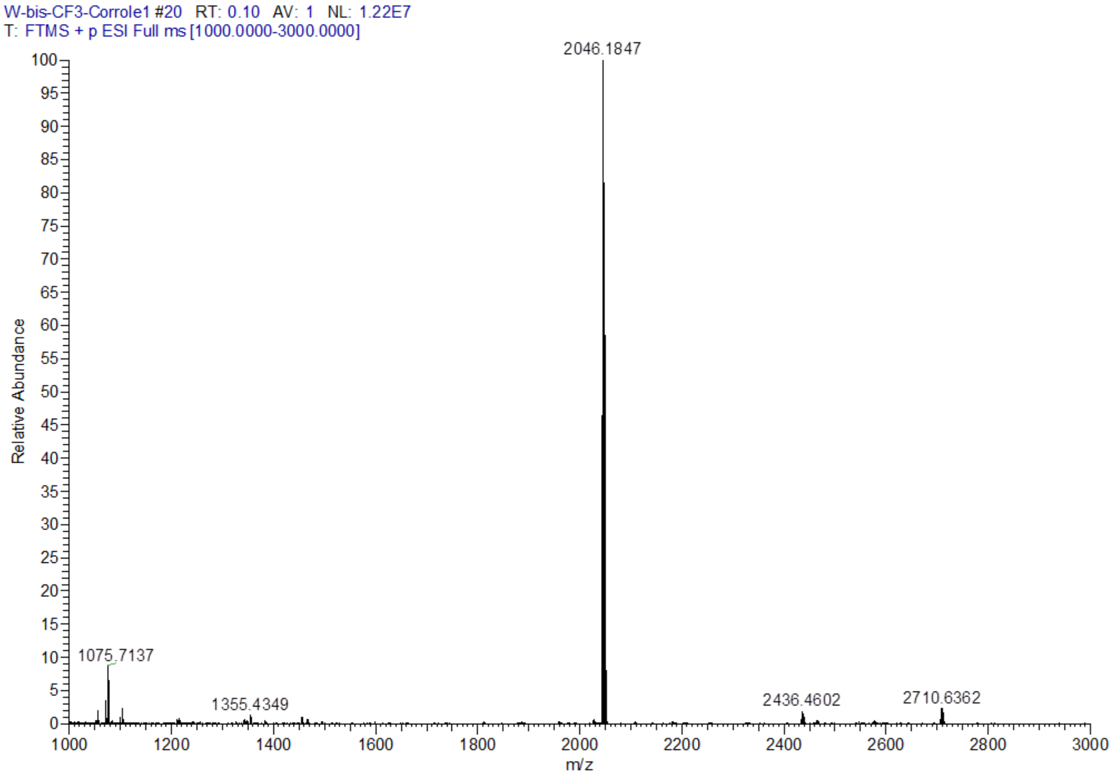

Figure S7. HRMS of D135-W[TBCF$_3$PC]$_2$.

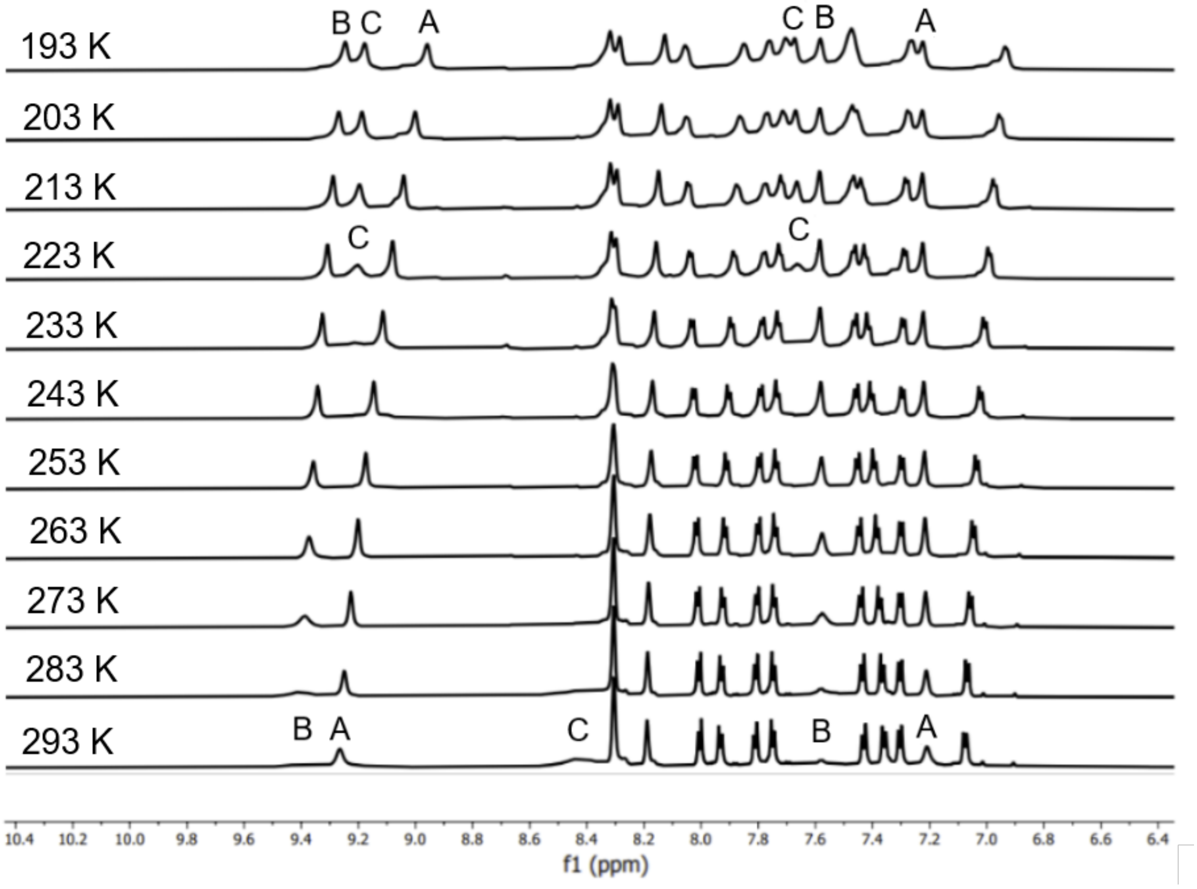

Figure S8. Variable-temperature ^{1}H NMR spectra of D135-W[TBCF$_3$PC]$_2$ in dichloromethane-d_2 from 193 K (top) to 293 K (bottom) in increments of 10 K. Group A peaks: 10-*o*-aryl exhibiting slow equilibrium; Group B: 5,15-*o*-aryl exhibiting coalescence; group C: 5,15-*o*-Ph exhibiting fast equilibrium.

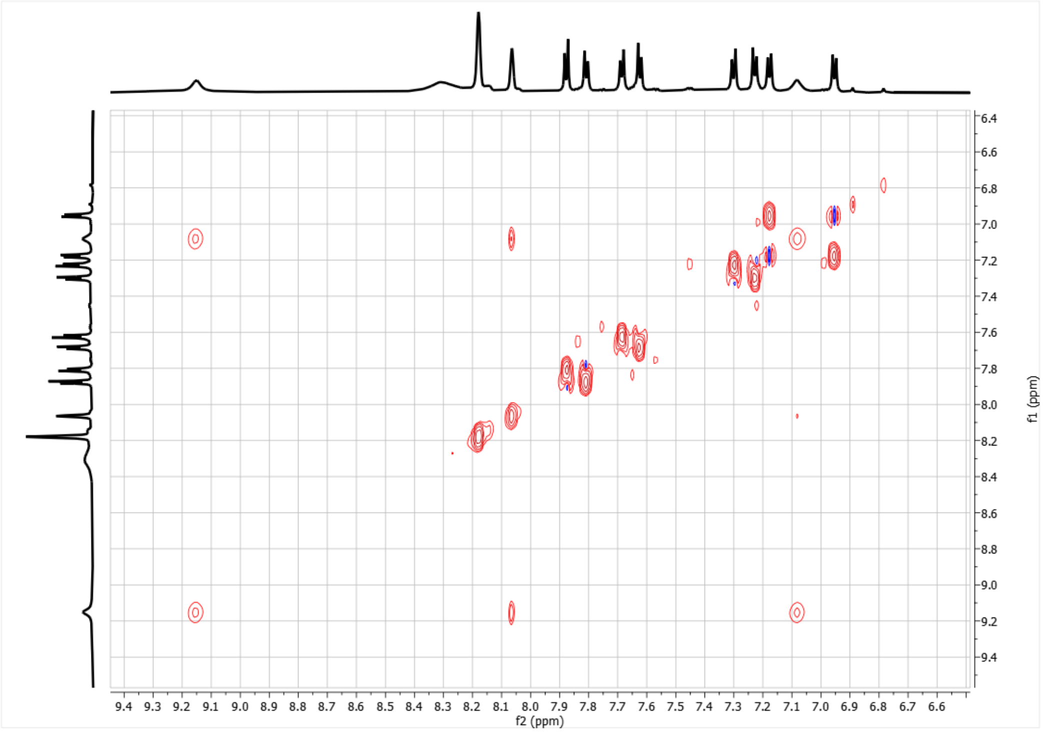

Figure S9. [1]H-^{1}H TOCSY of D135-W[TBCF$_3$PC]$_2$ in dichloromethane-d_2.

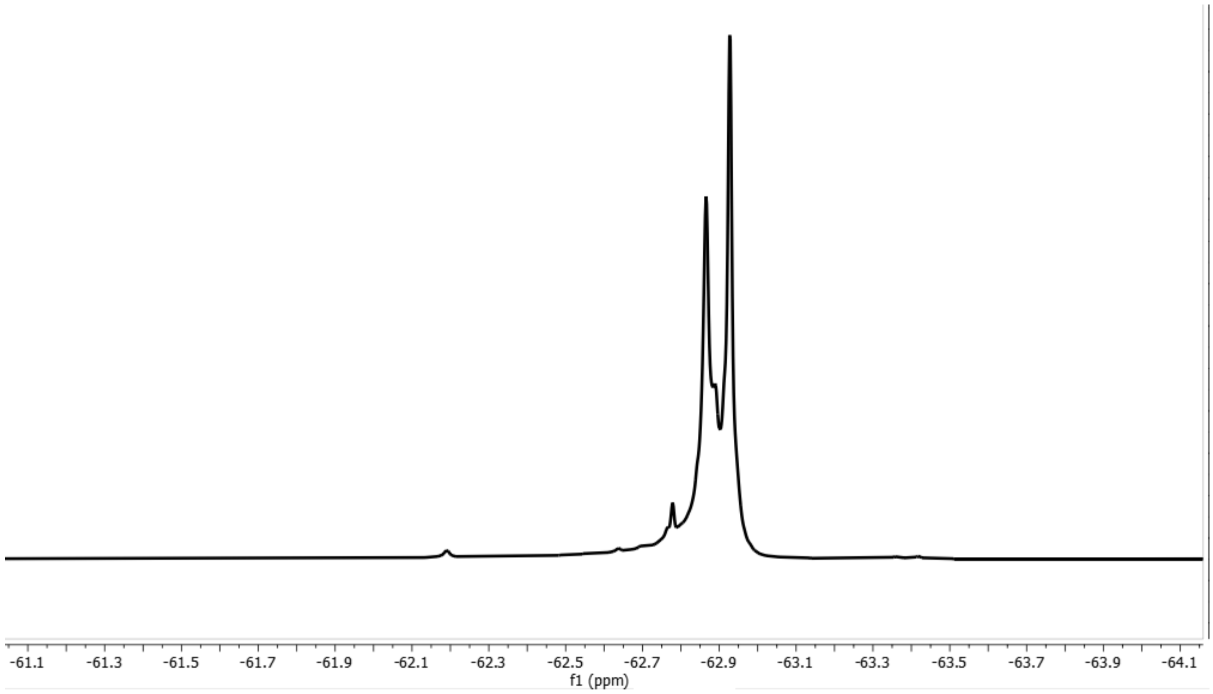

Figure S10. ^{19}F NMR spectrum of D135-W[TBCF$_3$PC]$_2$ in dichloromethane-d_2 at room temperature.

D45-W[TBCF$_3$PC]$_2$

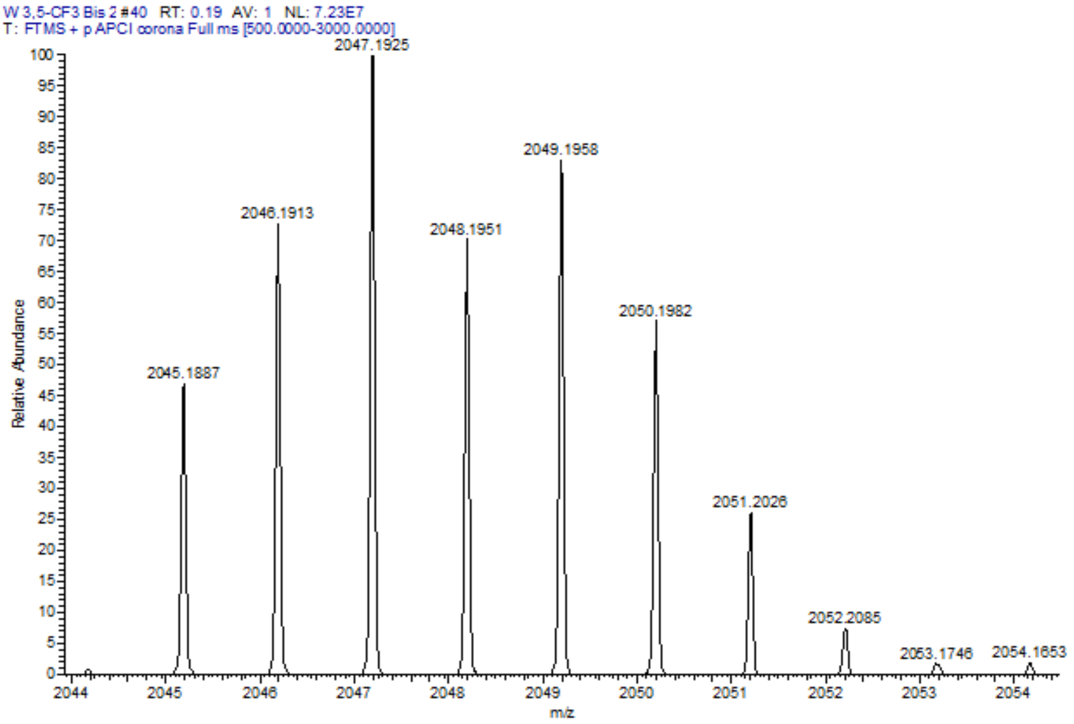

Figure S11. HRMS (APCI) of D45-W[TBCF$_3$PC]$_2$ with M+1 peak resulting from protonation.

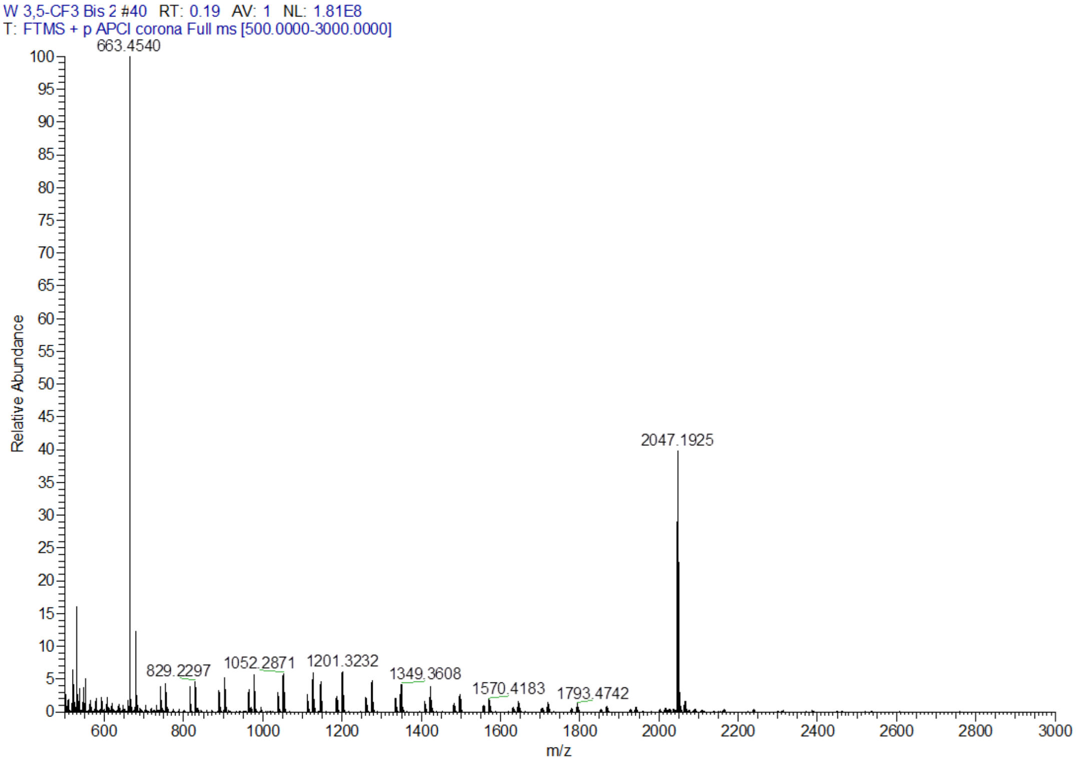

Figure S12. HRMS (APCI) of D45-W[TBCF$_3$PC]$_2$ with M+1 peak resulting from protonation.

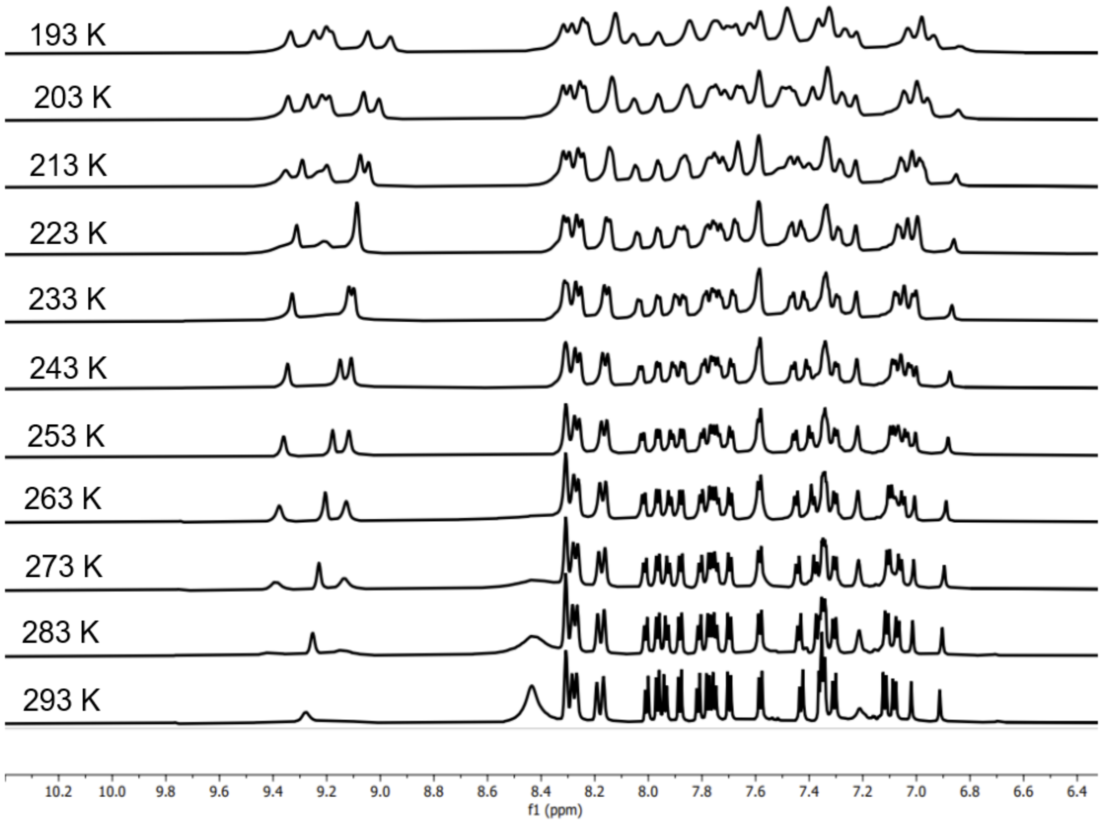

Figure S13. Variable temperature ^{1}H NMR spectra of D45-W[TBCF$_3$PC]$_2$ in dichloromethane-d_2 from 193 K (top) to 293 K (bottom) in increments of 10 K.

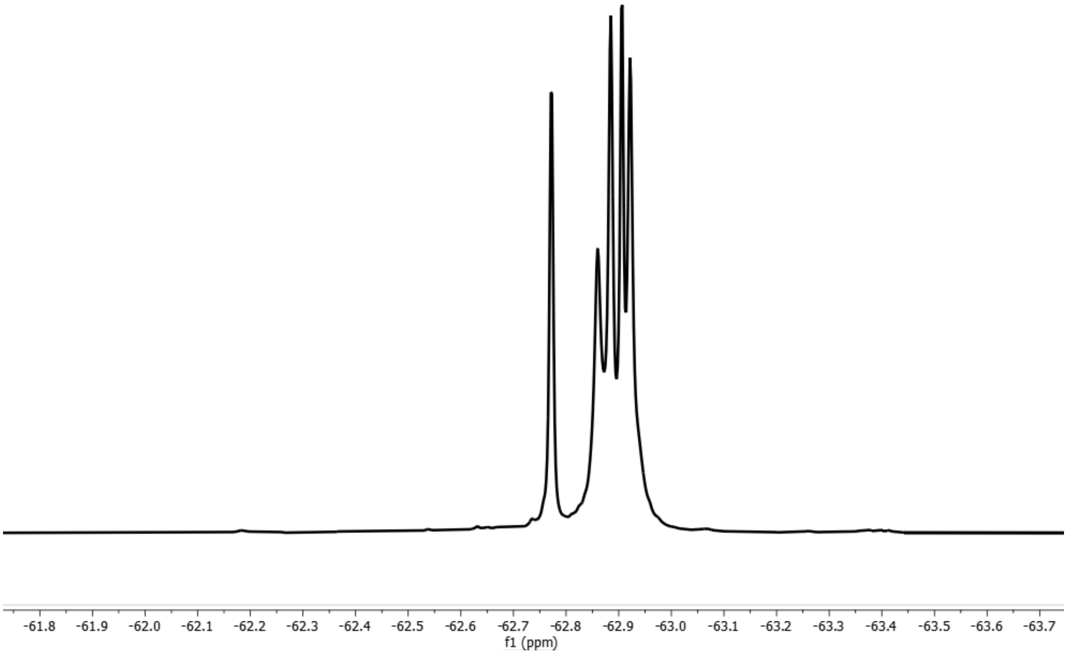

Figure S14. ^{19}F NMR spectrum of D45-W[TBCF$_3$PC]$_2$ in dichloromethane-d_2 at room temperature.

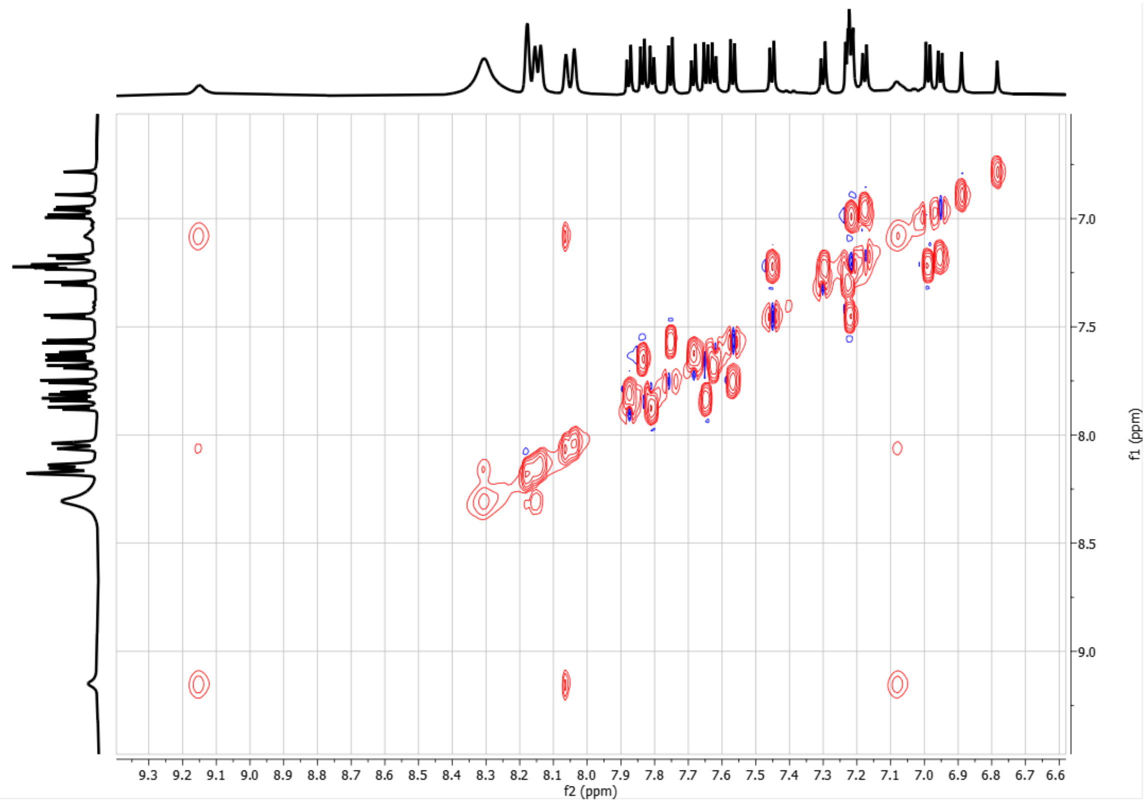

Figure S15. ^{1}H-^{1}H TOCSY of D45-W[TBCF_3PC]$_2$ in dichloromethane-d_2.

W[TDOMePC]2

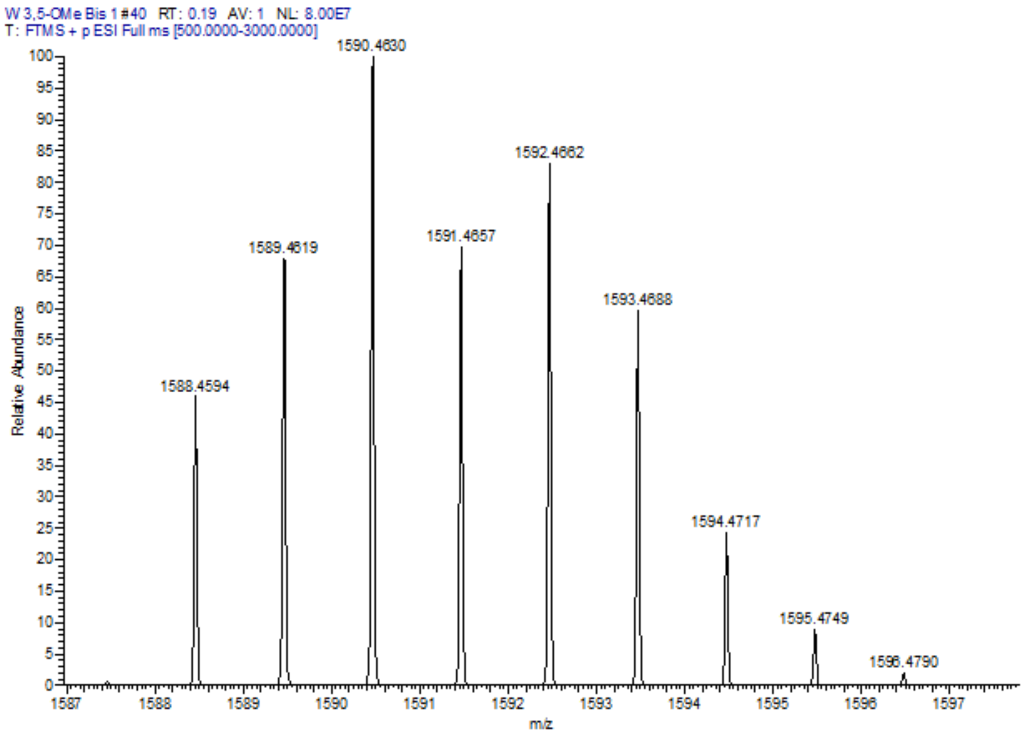

Figure S16. HRMS of D135-W[TDOMePC]$_2$.

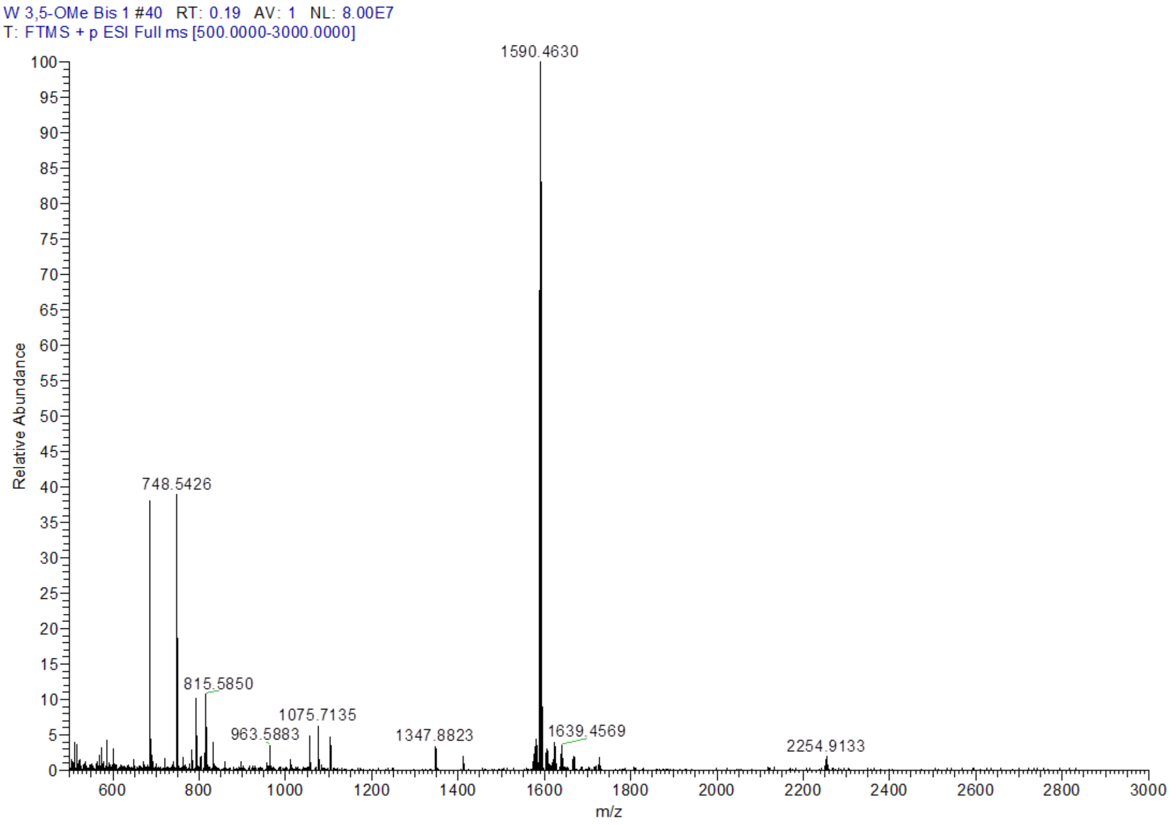

Figure S17. HRMS of D135-W[TDOMePC]$_2$.

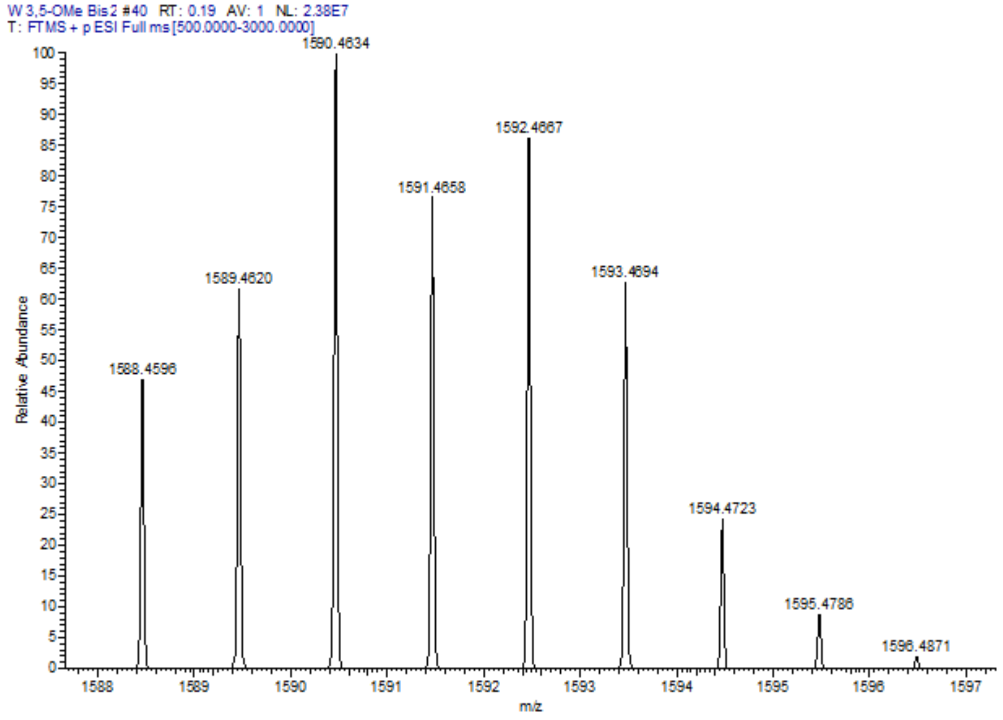

Figure S18. HRMS of D45-W[TDOMePC]$_2$.

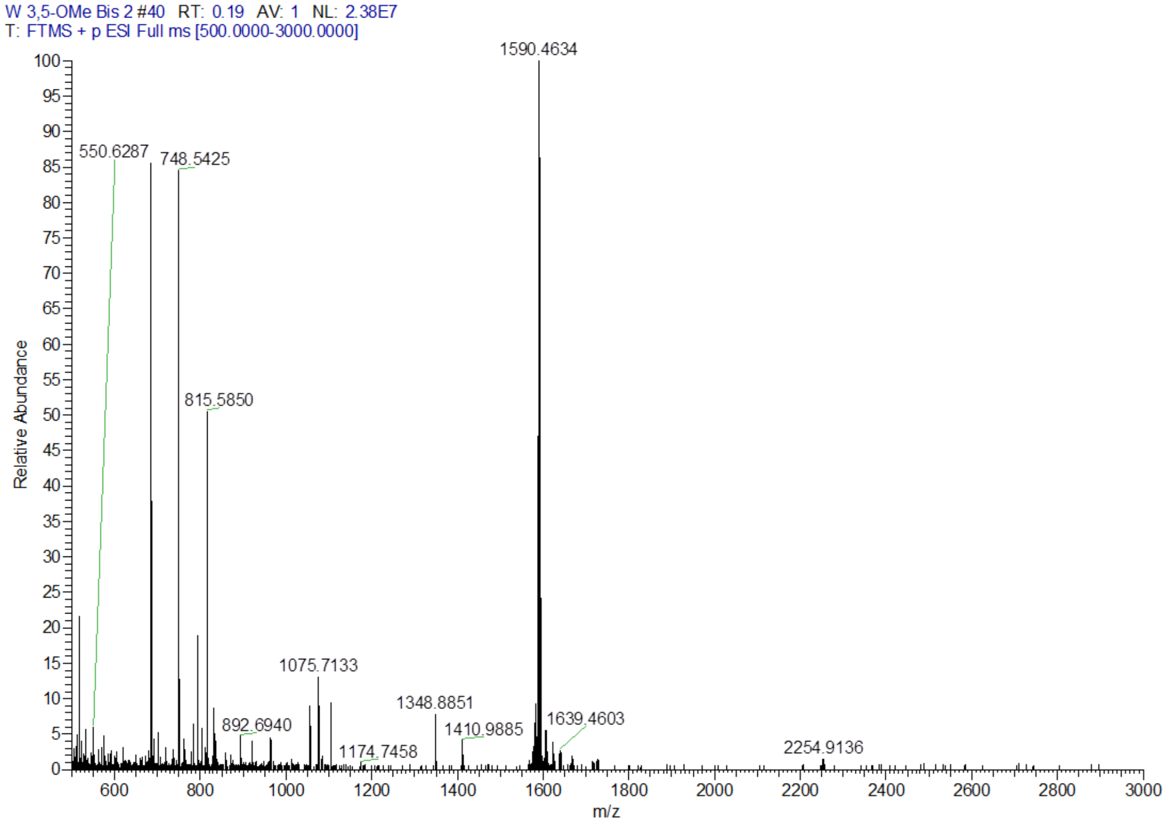

Figure S19. HRMS of D45-W[TDOMePC]$_2$.

Mo[TBCF$_3$PC]$_2$ (faster-moving isomer)

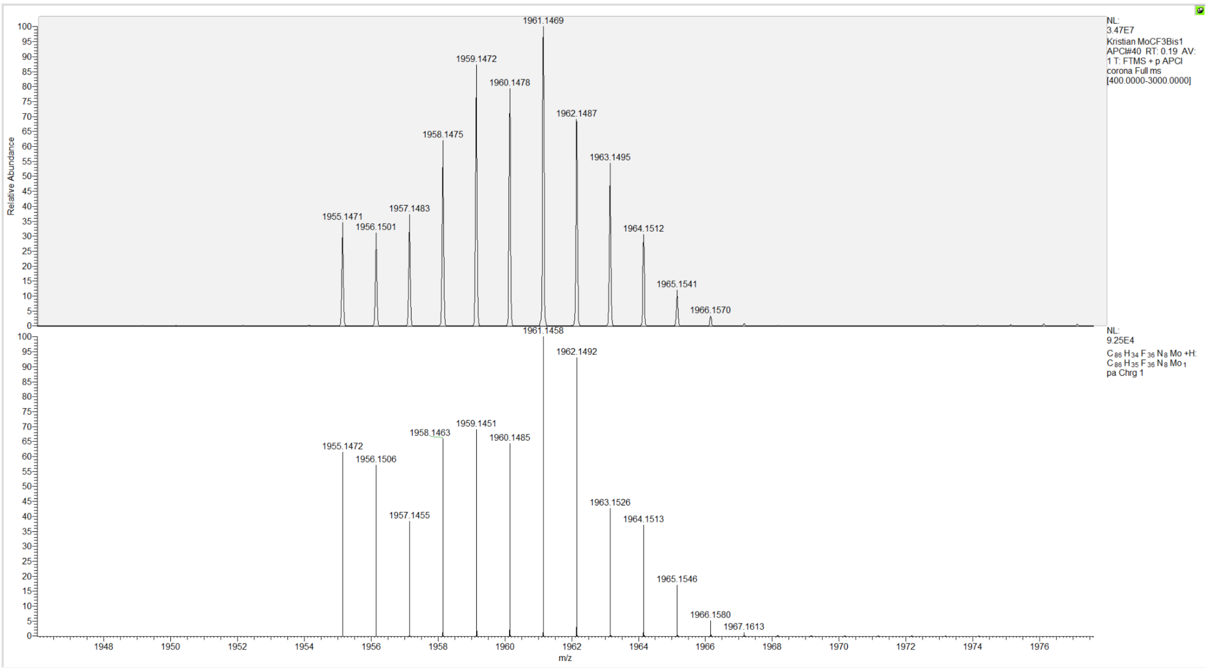

Figure S20. HRMS of the faster-moving isomer of Mo[TBCF$_3$PC]$_2$ (top) and theoretical simulation (bottom).

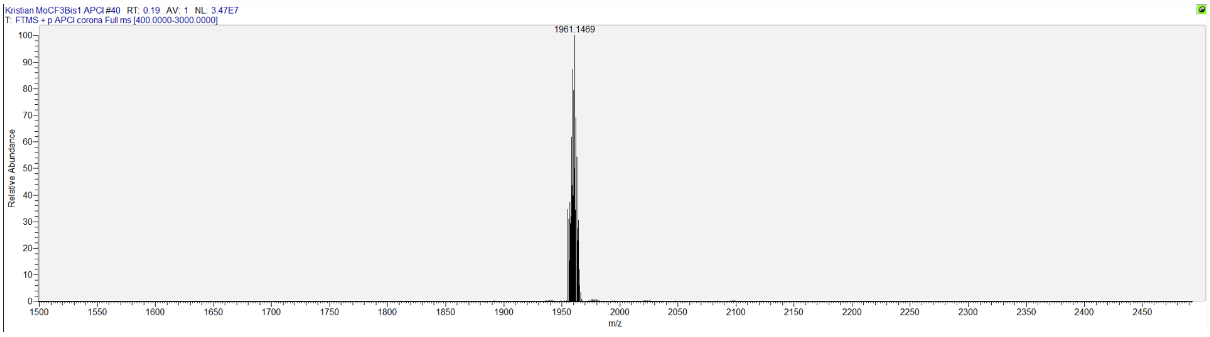

Figure S21. HRMS of the faster-moving isomer of Mo[TBCF$_3$PC]$_2$.

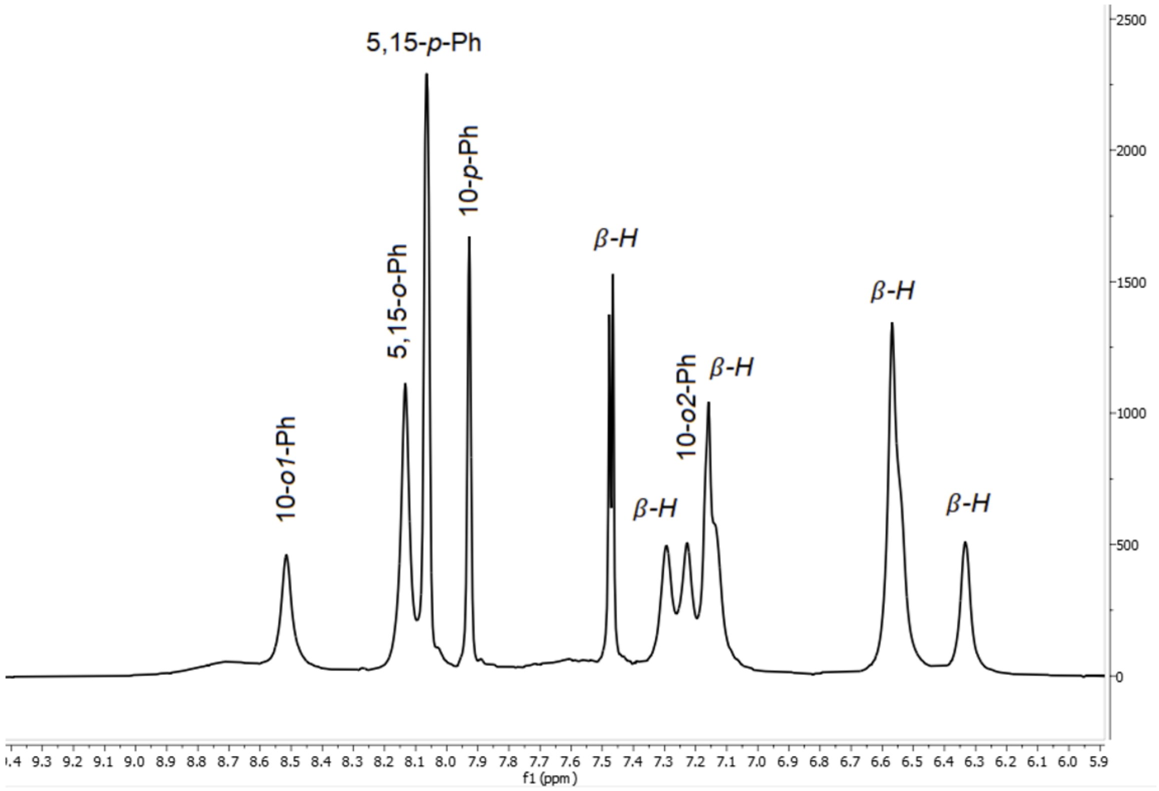

Figure S22. ¹H NMR spectrum of the faster-moving isomer of Mo[TBCF_3PC]$_2$ in dichloromethane-d_2 at room temperature.

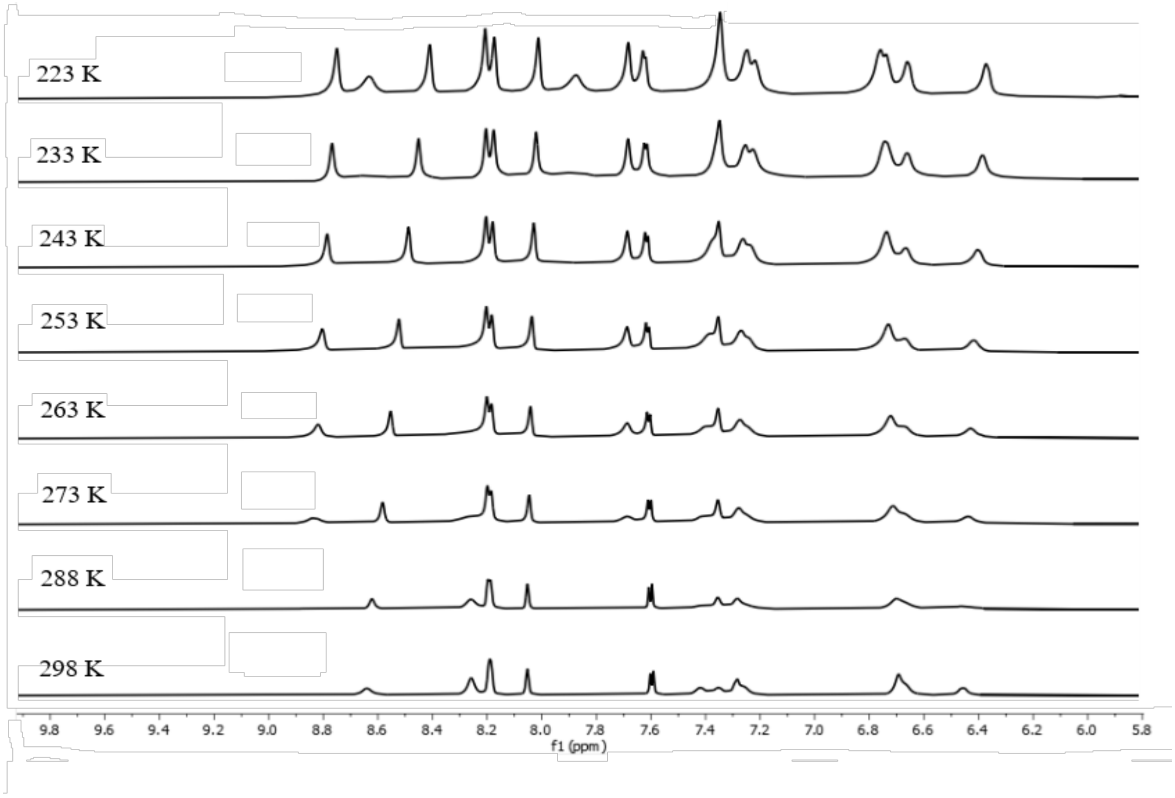

Figure S23. Variable temperature ¹H NMR spectra of the faster-moving isomer of Mo[TBCF_3PC]$_2$ in dichloromethane-d_2.

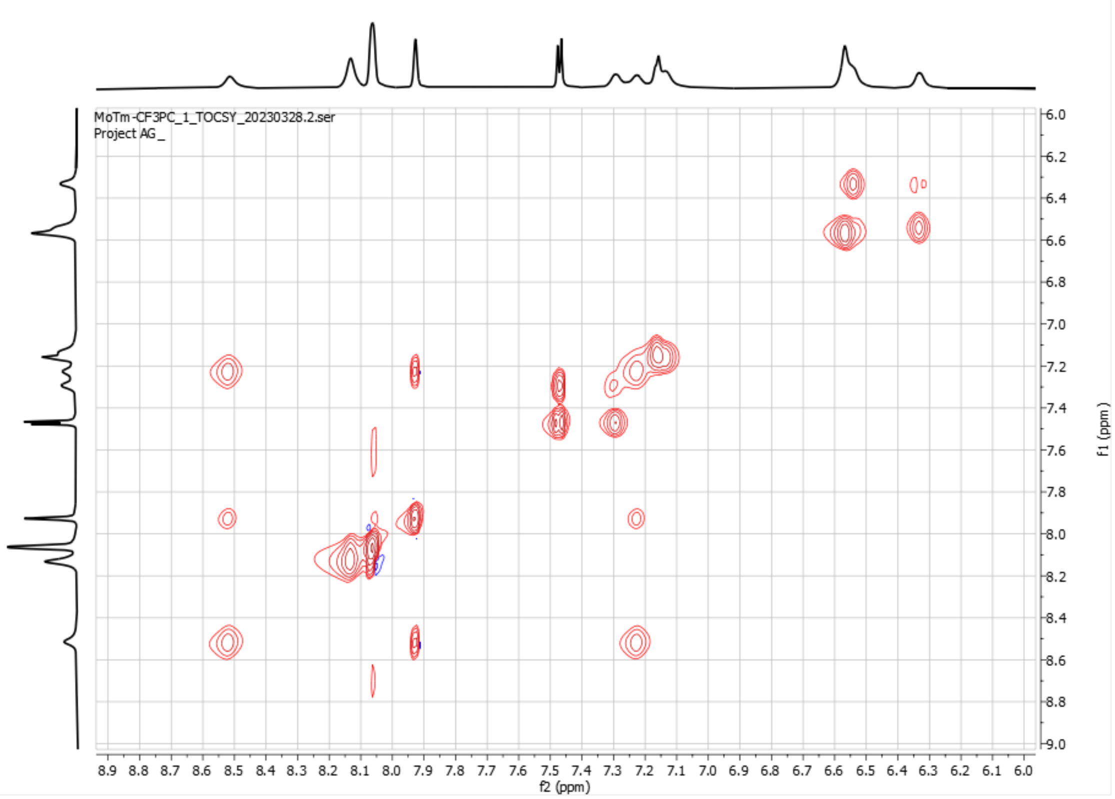

Figure S24. [1]H TOCSY of the faster-moving isomer of Mo[TBCF_3PC$]_2$ in dichloromethane-d_2.

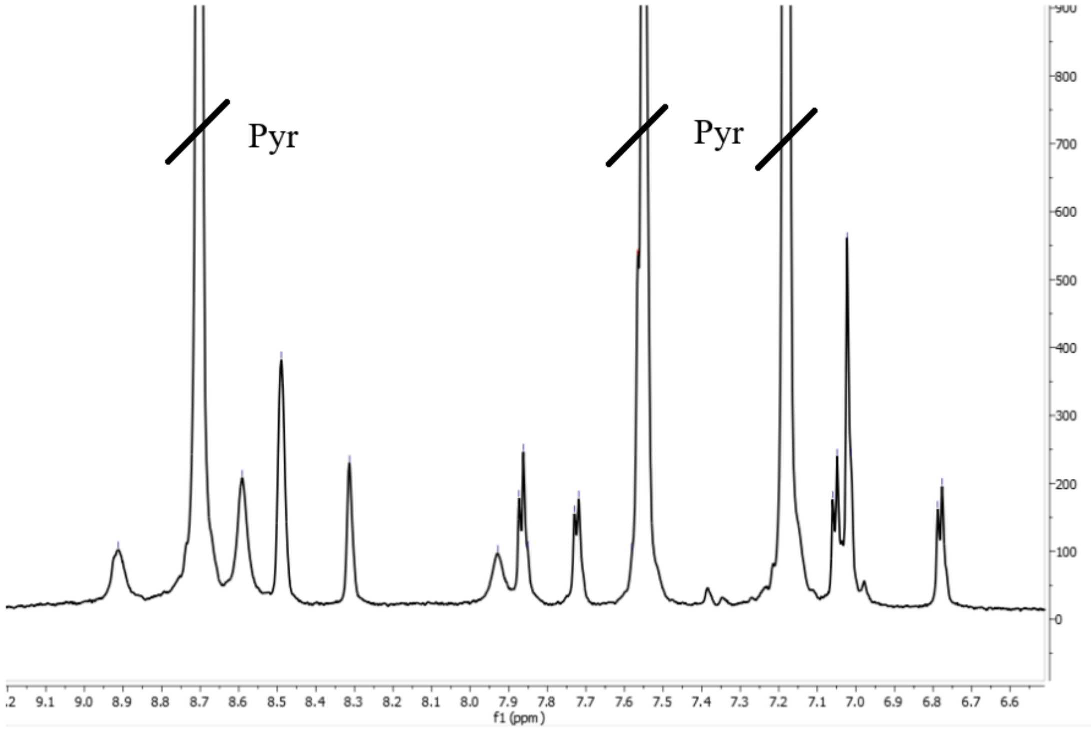

Figure S25. ^{1}H NMR spectrum of the faster-moving isomer of Mo[TBCF_3PC$]_2$ in pyridine-d_5 at room temperature.

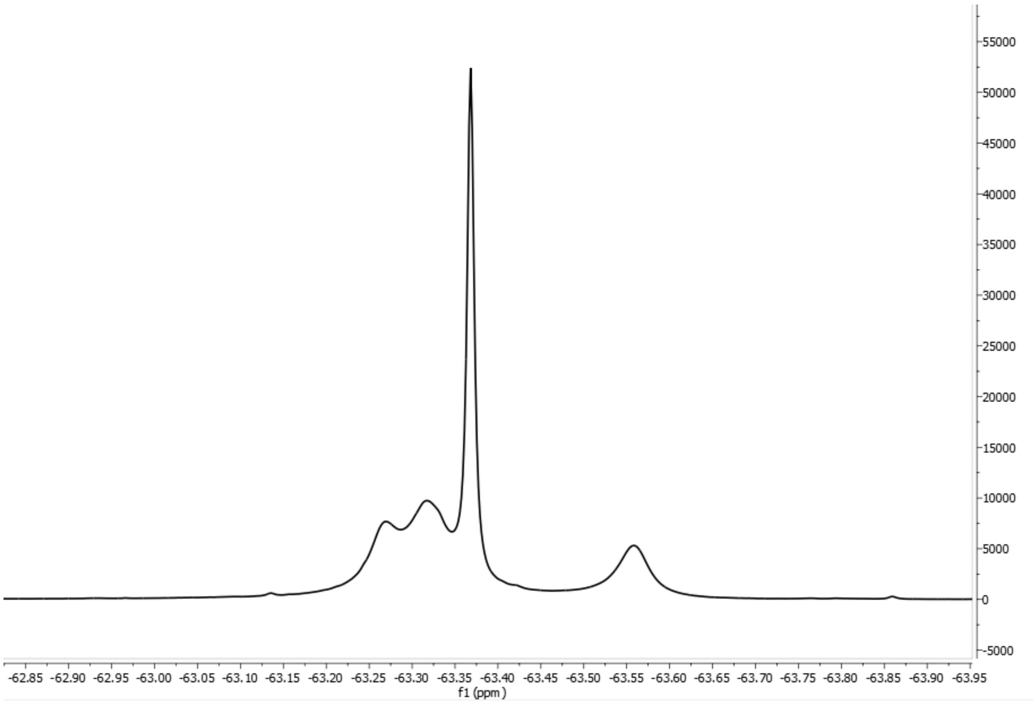

Figure S26. ^{19}F NMR spectrum of the faster-moving isomer of $Mo[TBCF_3PC]_2$ in dichloromethane-d_2 at room temperature.

Mo[TBCF$_3$PC]$_2$ (slower-moving isomer)

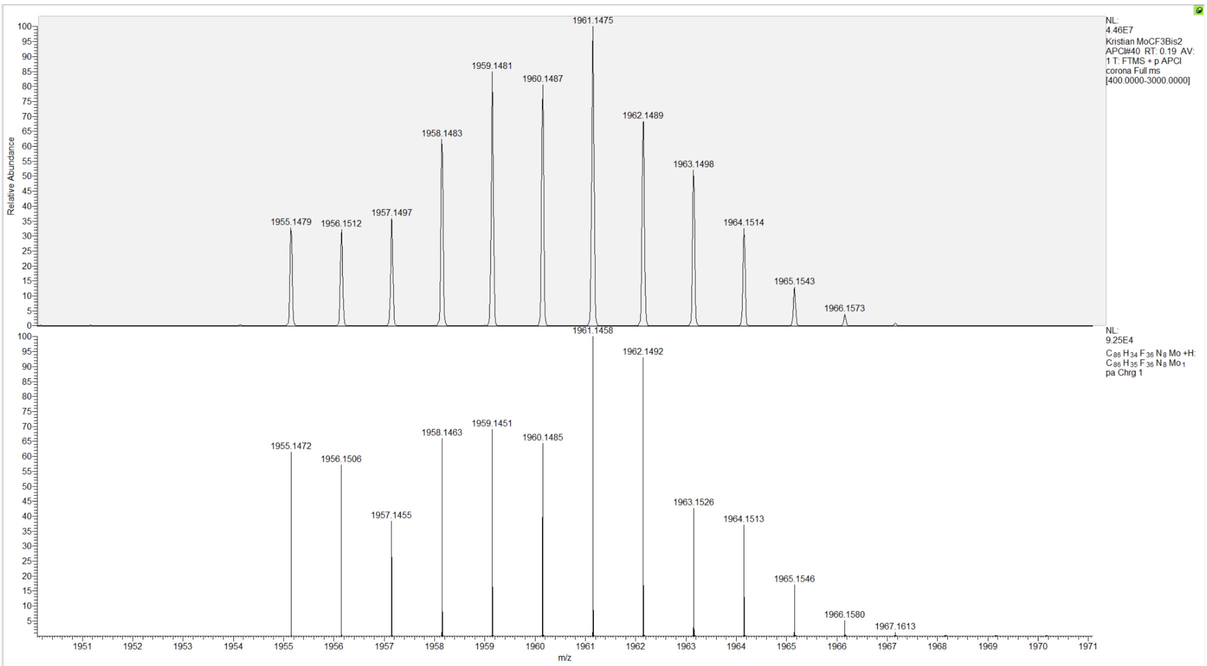

Figure S27. HRMS of the slower-moving isomer of Mo[TBCF$_3$PC]$_2$ (top) and theoretical mass spectrometry of Mo[T*m*-CF$_3$PC]$_2$ (bottom).

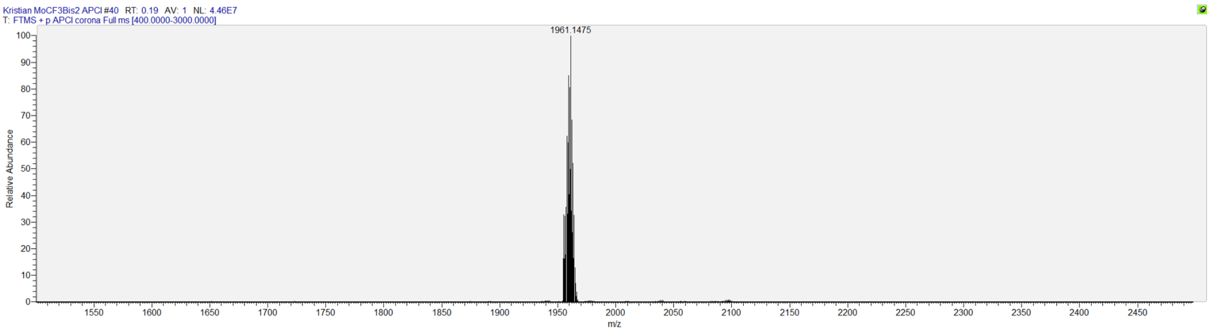

Figure S28. HRMS of the slower-moving isomer of Mo[TBCF$_3$PC]$_2$.

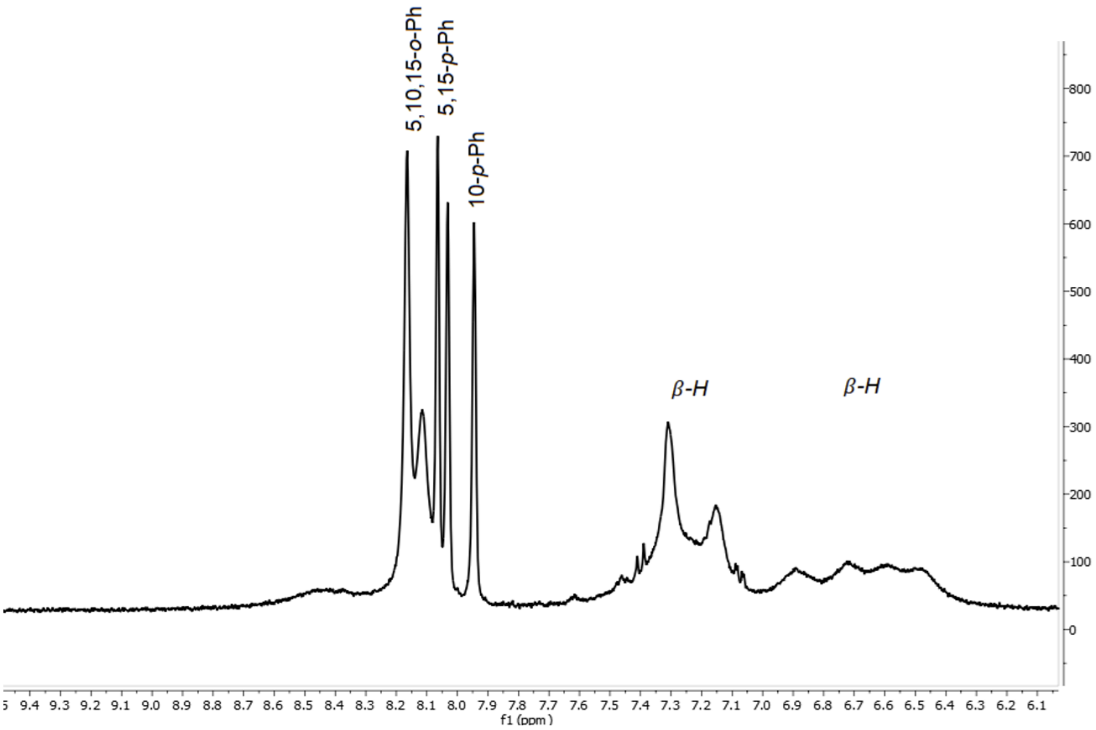

Figure S29. ¹H NMR spectrum of the slower-moving isomer of Mo[$TBCF_3PC$]$_2$ in dichloromethane-d_2 at room temperature.

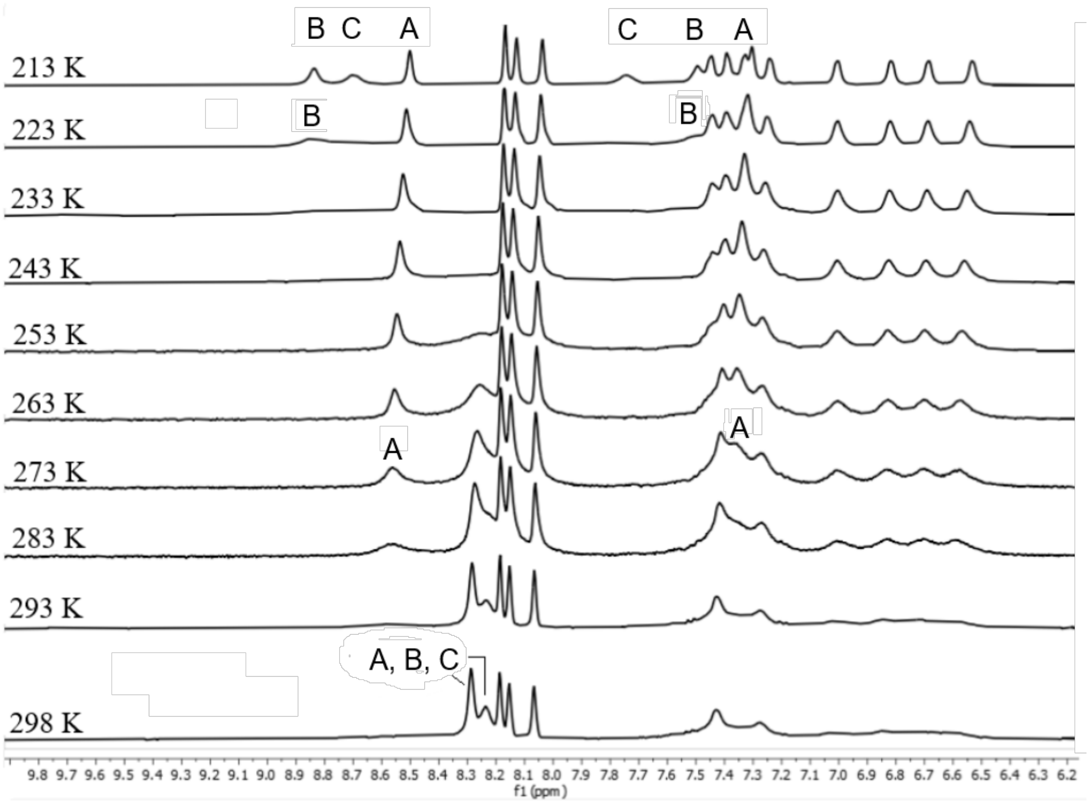

Figure S30. Variable temperature ¹H NMR of the slower-moving isomer of Mo[$TBCF_3PC$]$_2$ in dichloromethane-d_2. Group A, B, C mix of 5,10,15-*o*-Ph which all display fast equilibrium in room temperature.

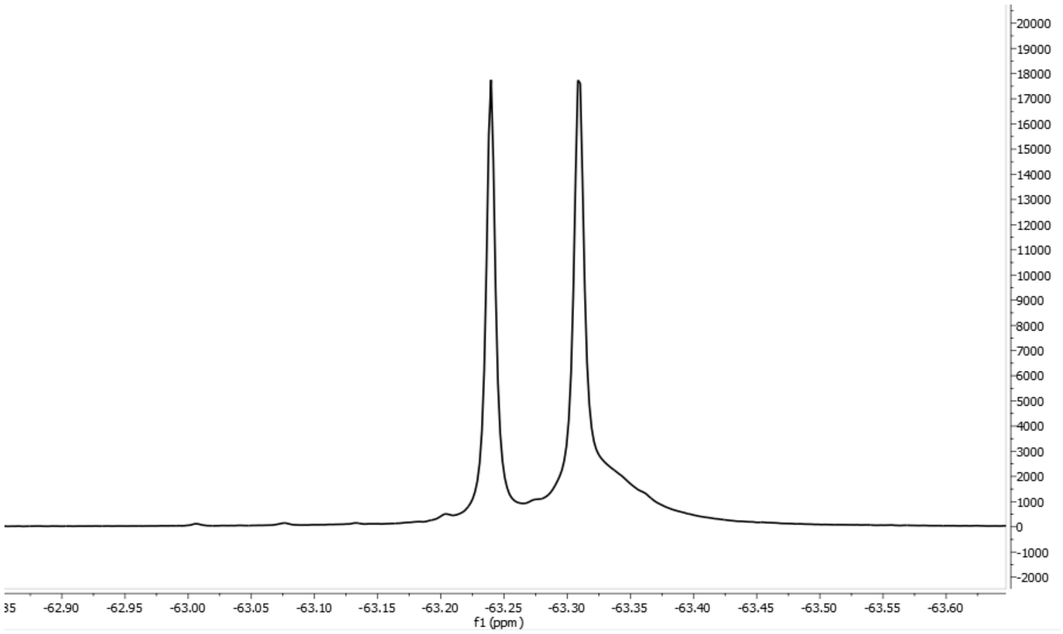

Figure S31. ^{19}F NMR spectrum of the slower-moving isomer of Mo[TBCF$_3$PC]$_2$ in dichloromethane-d_2 at room temperature.

OLYP-D3/ZORA-STO-TZ2P optimized coordinates (Å)

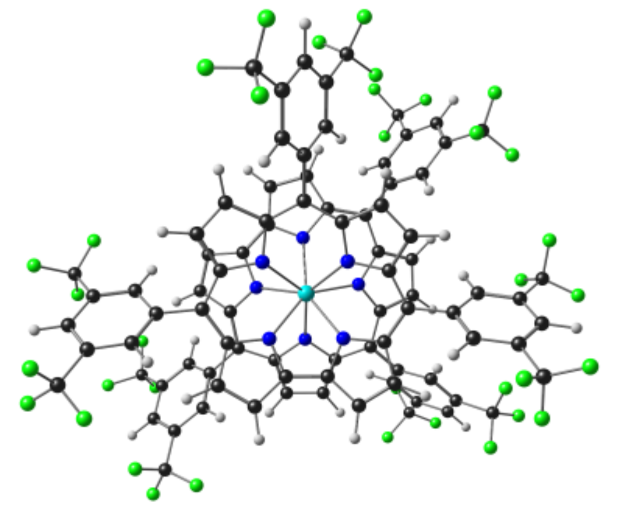	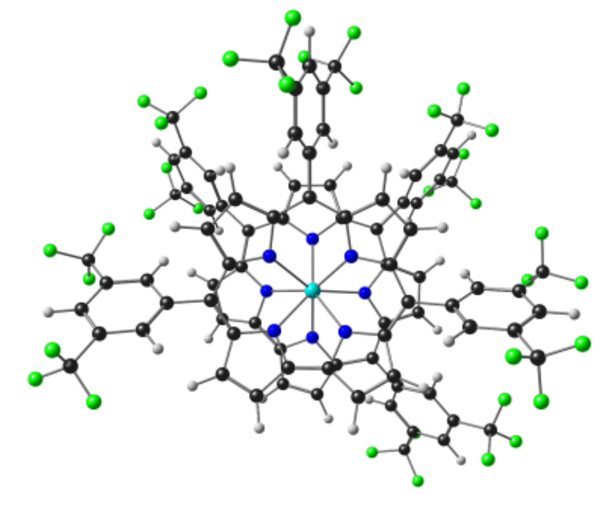
D135-W[TBCF$_3$PC]$_2$<br>ΔE = 0.01 eV	D45-W[TBCF$_3$PC]$_2$<br>ΔE = 0.00 eV

D135-W[TBCF_3PC]$_2$

74	0.000000000	0.000000000	0.470811000
7	0.498722000	1.313527000	2.124955000
7	0.834771000	-1.054017000	-1.298082000
7	-1.905471000	0.830470000	1.279422000
7	-1.483602000	-1.436527000	-0.140987000
7	-0.498722000	-1.313527000	2.124955000
7	-0.834771000	1.054017000	-1.298082000
7	1.905471000	-0.830470000	1.279422000
7	1.483602000	1.436527000	-0.140987000
6	2.031234000	2.486618000	3.360453000
6	2.178084000	-1.162009000	-1.642612000
6	-1.724017000	-1.935815000	2.095324000
6	-3.264971000	0.955614000	-0.787905000
6	2.302929000	-1.627129000	-2.986556000
6	0.959989000	2.195419000	4.181484000
6	-0.015802000	1.504013000	3.396930000
6	-2.284695000	-1.991996000	0.825053000
6	-3.120241000	0.835520000	0.595339000
6	0.122788000	-1.470900000	-2.428277000
6	1.036510000	-1.809700000	-3.471352000
6	-2.233578000	0.931986000	2.634178000
6	-1.347465000	1.201993000	3.691122000
6	-2.018204000	-1.803701000	-1.361401000
6	-3.401428000	-2.633054000	0.233764000
6	-4.201762000	0.843418000	1.530694000
6	-1.253850000	-1.735686000	-2.519444000
6	-3.656144000	0.917807000	2.781356000
6	-3.250868000	-2.496467000	-1.130965000
6	-2.031234000	-2.486618000	3.360453000
6	-2.178084000	1.162009000	-1.642612000
6	1.724017000	1.935815000	2.095324000
6	3.264971000	-0.955614000	-0.787905000
6	-2.302929000	1.627129000	-2.986556000
6	-0.959989000	-2.195419000	4.181484000
6	0.015802000	-1.504013000	3.396930000
6	2.284695000	1.991996000	0.825053000
6	3.120241000	-0.835520000	0.595339000
6	-0.122788000	1.470900000	-2.428277000
6	-1.036510000	1.809700000	-3.471352000
6	2.233578000	-0.931986000	2.634178000
6	1.347465000	-1.201993000	3.691122000
6	2.018204000	1.803701000	-1.361401000
6	3.401428000	2.633054000	0.233764000
6	4.201762000	-0.843418000	1.530694000
6	1.253850000	1.735686000	-2.519444000
6	3.656144000	-0.917807000	2.781356000
6	3.250868000	2.496467000	-1.130965000
1	-2.927508000	-3.036601000	3.612155000
1	3.238712000	-1.834436000	-3.481581000
1	0.853794000	2.463324000	5.219335000
1	-4.184684000	-3.154804000	0.766065000
1	0.743257000	-2.203190000	-4.433277000
1	-5.247626000	0.843297000	1.268199000
1	-4.175901000	1.014944000	3.721898000
1	-3.884841000	-2.906104000	-1.901694000
1	2.927508000	3.036601000	3.612155000
1	-3.238712000	1.834436000	-3.481581000
1	-0.853794000	-2.463324000	5.219335000
1	4.184684000	3.154804000	0.766065000
1	-0.743257000	2.203190000	-4.433277000
1	5.247626000	-0.843297000	1.268199000
1	4.175901000	-1.014944000	3.721898000
1	3.884841000	2.906104000	-1.901694000
6	-1.852058000	1.280511000	5.087749000
6	-2.560270000	0.201255000	5.639061000
6	-1.613126000	2.396877000	5.905322000
6	-2.964657000	0.211573000	6.972849000
6	-1.995780000	2.394905000	7.247416000
6	-2.669275000	1.300884000	7.793946000
1	-2.773658000	-0.656007000	5.019194000
1	-1.126810000	3.271510000	5.493314000
1	-2.959935000	1.299611000	8.838365000
6	1.841249000	2.173203000	-3.815485000
6	1.947012000	1.284329000	-4.891765000
6	2.314649000	3.483500000	-3.976847000
6	2.551588000	1.682514000	-6.085553000
6	2.904713000	3.882510000	-5.176194000
6	3.035642000	2.981851000	-6.235112000
1	1.585680000	0.271741000	-4.775844000
1	2.211506000	4.191507000	-3.162816000
1	3.509489000	3.288488000	-7.159800000
6	-4.632869000	1.029259000	-1.377748000
6	-5.030842000	0.118388000	-2.367781000
6	-5.534302000	2.029048000	-0.990369000

6	-6.309759000	0.181589000	-2.920449000
6	-6.811996000	2.094506000	-1.551448000
6	-7.213482000	1.163574000	-2.508185000
1	-4.332510000	-0.634931000	-2.702514000
1	-5.228771000	2.767144000	-0.257598000
1	-8.205367000	1.214266000	-2.941512000
6	-1.841249000	-2.173203000	-3.815485000
6	-2.314649000	-3.483500000	-3.976847000
6	-1.947012000	-1.284329000	-4.891765000
6	-2.904713000	-3.882510000	-5.176194000
6	-2.551588000	-1.682514000	-6.085553000
6	-3.035642000	-2.981851000	-6.235112000
1	-2.211506000	-4.191507000	-3.162816000
1	-1.585680000	-0.271741000	-4.775844000
1	-3.509489000	-3.288488000	-7.159800000
6	1.852058000	-1.280511000	5.087749000
6	1.613126000	-2.396877000	5.905322000
6	2.560270000	-0.201255000	5.639061000
6	1.995780000	-2.394905000	7.247416000
6	2.964657000	-0.211573000	6.972849000
6	2.669275000	-1.300884000	7.793946000
1	1.126810000	-3.271510000	5.493314000
1	2.773658000	0.656007000	5.019194000
1	2.959935000	-1.299611000	8.838365000
6	4.632869000	-1.029259000	-1.377748000
6	5.534302000	-2.029048000	-0.990369000
6	5.030842000	-0.118388000	-2.367781000
6	6.811996000	-2.094506000	-1.551448000
6	6.309759000	-0.181589000	-2.920449000
6	7.213482000	-1.163574000	-2.508185000
1	5.228771000	-2.767144000	-0.257598000
1	4.332510000	0.634931000	-2.702514000
1	8.205367000	-1.214266000	-2.941512000
6	3.460679000	5.287376000	-5.303615000
9	2.725272000	6.185360000	-4.604104000
9	4.731832000	5.354336000	-4.822416000
9	3.498604000	5.700043000	-6.590118000
6	2.660865000	0.687446000	-7.224240000
9	1.486153000	0.580764000	-7.897585000
9	3.611548000	1.038699000	-8.118119000
9	2.967456000	-0.556395000	-6.769996000
6	3.654097000	1.005358000	7.558253000
9	4.319970000	1.715449000	6.613810000
9	2.750984000	1.851413000	8.127126000
9	4.544394000	0.669227000	8.518522000
6	1.700625000	-3.598040000	8.123066000
9	2.764155000	-4.440983000	8.182261000
9	1.412921000	-3.226234000	9.392953000
9	0.647729000	-4.315026000	7.660342000
6	6.752945000	0.855947000	-3.931966000
9	7.624518000	0.347093000	-4.833029000
9	5.707230000	1.369935000	-4.620971000
9	7.376017000	1.897625000	-3.315926000
6	7.763873000	-3.176183000	-1.083596000
9	8.806627000	-3.340037000	-1.927171000
9	8.276473000	-2.876869000	0.141165000
9	7.136495000	-4.371868000	-0.966949000
6	-7.763873000	3.176183000	-1.083596000
9	-8.806627000	3.340037000	-1.927171000
9	-8.276473000	2.876869000	0.141165000
9	-7.136495000	4.371868000	-0.966949000
6	-6.752945000	-0.855947000	-3.931966000
9	-7.624518000	-0.347093000	-4.833029000
9	-5.707230000	-1.369935000	-4.620971000
9	-7.376017000	-1.897625000	-3.315926000
6	-2.660865000	-0.687446000	-7.224240000
9	-1.486153000	-0.580764000	-7.897585000
9	-3.611548000	-1.038699000	-8.118119000
9	-2.967456000	0.556395000	-6.769996000
6	-3.460679000	-5.287376000	-5.303615000
9	-2.725272000	-6.185360000	-4.604104000
9	-4.731832000	-5.354336000	-4.822416000
9	-3.498604000	-5.700043000	-6.590118000
6	-1.700625000	3.598040000	8.123066000
9	-2.764155000	4.440983000	8.182261000
9	-1.412921000	3.226234000	9.392953000
9	-0.647729000	4.315026000	7.660342000
6	-3.654097000	-1.005358000	7.558253000
9	-4.319970000	-1.715449000	6.613810000
9	-2.750984000	-1.851413000	8.127126000
9	-4.544394000	-0.669227000	8.518522000

D45-W[TBCF_3PC]$_2$

74	0.000000000	0.000000000	-1.150463000
7	0.455964000	1.248916000	-2.868152000
7	0.967421000	-1.020900000	0.547823000
7	1.595705000	1.435537000	-0.550597000
7	1.790679000	-0.933989000	-1.936288000
7	-0.455964000	-1.248916000	-2.868152000
7	-0.967421000	1.020900000	0.547823000
7	-1.595705000	-1.435537000	-0.550597000
7	-1.790679000	0.933989000	-1.936288000
6	0.019427000	1.810578000	-5.047311000
6	0.335794000	-1.467414000	1.707663000
6	0.535380000	-1.436408000	-3.799042000
6	1.019005000	1.764482000	1.842900000
6	1.307780000	-1.775202000	2.706514000
6	1.385312000	1.871826000	-4.863967000
6	1.651077000	1.587268000	-3.483536000
6	1.809069000	-1.293234000	-3.262450000
6	1.889596000	1.829491000	0.756062000
6	2.338193000	-1.104583000	0.814418000
6	2.539604000	-1.541865000	2.159875000
6	2.686953000	1.851066000	-1.318771000
6	2.790926000	1.836071000	-2.723657000
6	3.094849000	-1.037453000	-1.479755000
6	3.133442000	-1.558744000	-3.689395000
6	3.176722000	2.444267000	0.802586000
6	3.385071000	-1.028474000	-0.119937000
6	3.662690000	2.464336000	-0.474930000
6	3.939873000	-1.381404000	-2.584584000
6	-0.019427000	-1.810578000	-5.047311000
6	-0.335794000	1.467414000	1.707663000
6	-0.535380000	1.436408000	-3.799042000
6	-1.019005000	-1.764482000	1.842900000
6	-1.307780000	1.775202000	2.706514000
6	-1.385312000	-1.871826000	-4.863967000
6	-1.651077000	-1.587268000	-3.483536000
6	-1.809069000	1.293234000	-3.262450000
6	-1.889596000	-1.829491000	0.756062000
6	-2.338193000	1.104583000	0.814418000
6	-2.539604000	1.541865000	2.159875000
6	-2.686953000	-1.851066000	-1.318771000
6	-2.790926000	-1.836071000	-2.723657000
6	-3.094849000	1.037453000	-1.479755000
6	-3.133442000	1.558744000	-3.689395000
6	-3.176722000	-2.444267000	0.802586000
6	-3.385071000	1.028474000	-0.119937000
6	-3.662690000	-2.464336000	-0.474930000
6	-3.939873000	1.381404000	-2.584584000
1	0.538086000	-2.014848000	-5.951073000
1	1.076375000	-2.182205000	3.679061000
1	2.126671000	2.145147000	-5.598183000
1	3.427254000	-1.869291000	-4.682732000
1	3.506409000	-1.722090000	2.604183000
1	3.632850000	2.857314000	1.688261000
1	4.583386000	2.906496000	-0.822324000
1	5.005662000	-1.536675000	-2.524818000
1	-0.538086000	2.014848000	-5.951073000
1	-1.076375000	2.182205000	3.679061000
1	-2.126671000	-2.145147000	-5.598183000
1	-3.427254000	1.869291000	-4.682732000
1	-3.506409000	1.722090000	2.604183000
1	-3.632850000	-2.857314000	1.688261000
1	-4.583386000	-2.906496000	-0.822324000
1	-5.005662000	1.536675000	-2.524818000
6	4.057789000	2.264585000	-3.368962000
6	5.275795000	1.654214000	-3.029153000
6	4.078026000	3.292420000	-4.324592000
6	6.465082000	2.034495000	-3.650088000
6	5.263877000	3.649566000	-4.968389000
6	6.465892000	3.022019000	-4.638553000
1	5.283002000	0.879387000	-2.276263000
1	3.163191000	3.820804000	-4.561005000
1	7.387378000	3.303112000	-5.135187000
6	-4.787290000	1.167622000	0.354434000
6	-5.337780000	0.223425000	1.230090000
6	-5.573821000	2.265962000	-0.026255000
6	-6.648692000	0.357053000	1.691071000
6	-6.883623000	2.396855000	0.436007000
6	-7.433109000	1.438806000	1.291604000
1	-4.735115000	-0.619936000	1.538364000
1	-5.148755000	3.030682000	-0.665448000
1	-8.450750000	1.541407000	1.648354000
6	1.510709000	2.186329000	3.190973000
6	1.637490000	1.235600000	4.210842000
6	1.835539000	3.518991000	3.470890000

6	2.110802000	1.597735000	5.470874000
6	2.295238000	3.883654000	4.740394000
6	2.448493000	2.924965000	5.742513000
1	1.376110000	0.208768000	4.004900000
1	1.719918000	4.271781000	2.698858000
1	2.810272000	3.211185000	6.723187000
6	4.787290000	-1.167622000	0.354434000
6	5.573821000	-2.265962000	-0.026255000
6	5.337780000	-0.223425000	1.230090000
6	6.883623000	-2.396855000	0.436007000
6	6.648692000	-0.357053000	1.691071000
6	7.433109000	-1.438806000	1.291604000
1	5.148755000	-3.030682000	-0.665448000
1	4.735115000	0.619936000	1.538364000
1	8.450750000	-1.541407000	1.648354000
6	-4.057789000	-2.264585000	-3.368962000
6	-4.078026000	-3.292420000	-4.324592000
6	-5.275795000	-1.654214000	-3.029153000
6	-5.263877000	-3.649566000	-4.968389000
6	-6.465082000	-2.034495000	-3.650088000
6	-6.465892000	-3.022019000	-4.638553000
1	-3.163191000	-3.820804000	-4.561005000
1	-5.283002000	-0.879387000	-2.276263000
1	-7.387378000	-3.303112000	-5.135187000
6	-1.510709000	-2.186329000	3.190973000
6	-1.835539000	-3.518991000	3.470890000
6	-1.637490000	-1.235600000	4.210842000
6	-2.295238000	-3.883654000	4.740394000
6	-2.110802000	-1.597735000	5.470874000
6	-2.448493000	-2.924965000	5.742513000
1	-1.719918000	-4.271781000	2.698858000
1	-1.376110000	-0.208768000	4.004900000
1	-2.810272000	-3.211185000	6.723187000
6	-7.725124000	3.572755000	-0.022442000
9	-6.972500000	4.681975000	-0.221416000
9	-8.348430000	3.298357000	-1.200564000
9	-8.685729000	3.886817000	0.875776000
6	-7.186328000	-0.675677000	2.661952000
9	-6.621394000	-0.529264000	3.891382000
9	-8.523752000	-0.587730000	2.818893000
9	-6.905478000	-1.938752000	2.248568000
6	-7.760408000	-1.331354000	-3.292516000
9	-7.744209000	-0.863890000	-2.020269000
9	-7.981502000	-0.262881000	-4.106296000
9	-8.829453000	-2.151510000	-3.412527000
6	-5.244497000	-4.759281000	-6.002434000
9	-5.399040000	-5.980216000	-5.427225000
9	-6.236718000	-4.614473000	-6.910211000
9	-4.070021000	-4.788010000	-6.681829000
6	-2.331582000	-0.537473000	6.531613000
9	-2.038117000	-0.991768000	7.771465000
9	-1.579921000	0.569007000	6.310033000
9	-3.632097000	-0.137340000	6.549299000
6	-2.666773000	-5.328377000	5.012179000
9	-2.631511000	-5.622865000	6.331155000
9	-3.926108000	-5.600373000	4.574446000
9	-1.829665000	-6.184634000	4.377502000
6	2.666773000	5.328377000	5.012179000
9	2.631511000	5.622865000	6.331155000
9	3.926108000	5.600373000	4.574446000
9	1.829665000	6.184634000	4.377502000
6	2.331582000	0.537473000	6.531613000
9	2.038117000	0.991768000	7.771465000
9	1.579921000	-0.569007000	6.310033000
9	3.632097000	0.137340000	6.549299000
6	7.186328000	0.675677000	2.661952000
9	6.621394000	0.529264000	3.891382000
9	8.523752000	0.587730000	2.818893000
9	6.905478000	1.938752000	2.248568000
6	7.725124000	-3.572755000	-0.022442000
9	6.972500000	-4.681975000	-0.221416000
9	8.348430000	-3.298357000	-1.200564000
9	8.685729000	-3.886817000	0.875776000
6	5.244497000	4.759281000	-6.002434000
9	5.399040000	5.980216000	-5.427225000
9	6.236718000	4.614473000	-6.910211000
9	4.070021000	4.788010000	-6.681829000
6	7.760408000	1.331354000	-3.292516000
9	7.744209000	0.863890000	-2.020269000
9	7.981502000	0.262881000	-4.106296000
9	8.829453000	2.151510000	-3.412527000
